# Network Analysis Reveals a Common Host–Pathogen Interaction Pattern in *Arabidopsis* Immune Responses

**DOI:** 10.3389/fpls.2017.00893

**Published:** 2017-05-29

**Authors:** Hong Li, Yuan Zhou, Ziding Zhang

**Affiliations:** State Key Laboratory of Agrobiotechnology, College of Biological Sciences, China Agricultural UniversityBeijing, China

**Keywords:** effector, network analysis, plant immune response, plant–pathogen interaction, systems biology, time series gene expression data

## Abstract

Many plant pathogens secrete virulence effectors into host cells to target important proteins in host cellular network. However, the dynamic interactions between effectors and host cellular network have not been fully understood. Here, an integrative network analysis was conducted by combining *Arabidopsis thaliana* protein–protein interaction network, known targets of *Pseudomonas syringae* and *Hyaloperonospora arabidopsidis* effectors, and gene expression profiles in the immune response. In particular, we focused on the characteristic network topology of the effector targets and differentially expressed genes (DEGs). We found that effectors tended to manipulate key network positions with higher betweenness centrality. The effector targets, especially those that are common targets of an individual effector, tended to be clustered together in the network. Moreover, the distances between the effector targets and DEGs increased over time during infection. In line with this observation, pathogen-susceptible mutants tended to have more DEGs surrounding the effector targets compared with resistant mutants. Our results suggest a common plant–pathogen interaction pattern at the cellular network level, where pathogens employ potent local impact mode to interfere with key positions in the host network, and plant organizes an in-depth defense by sequentially activating genes distal to the effector targets.

## Introduction

Plants are under constant threat of a wide spectrum of pathogens including bacteria, oomycetes and fungi in the wild (Tsuda and Somssich, 2015). As a response, plants have evolved complicated immune systems against pathogens. Pathogen-associated molecular pattern (PAMP)-triggered immunity (PTI) and effector-triggered immunity (ETI) are two major phases of plant immunity (Thomma et al., 2011). Primarily, plants sense pathogens’ conserved PAMPs (e.g., flagellin) to trigger PTI. To subvert PTI, pathogens secrete a battery of effector proteins, which usually carry various enzymatic activities or enzyme inhibitor activities, to interfere with plant immunity (Dodds and Rathjen, 2010). As a counteraction, potent ETI can be activated on the recognition of these effectors by nucleotide binding-leucine rich repeat (NB-LRR) proteins, either directly (i.e., by interacting with the effectors directly) or indirectly (i.e., by interacting with the proteins attacked by the effectors) (Ye and Ting, 2008; Li and Zhang, 2016).

To investigate the mechanisms of plant–pathogen interactions, several model pathogens have been exploited, among which *Pseudomonas syringae (Psy)* and *Hyaloperonospora arabidopsidis (Hpa)* are two representative model pathogens that infect *Arabidopsis*. *Psy* is a bacterium which causes severe diseases in a wide range of plant species, and it is also the first model pathogen used for *Arabidopsis* (Katagiri et al., 2002). This pathogen undertakes an exceptional hemibiotrophic lifestyle during infection. In the early stage, *Psy* absorbs nutrients from living host cells for rapid multiplication. But in the late stage, it massively kills host cells, resulting in extensive necrosis of infected tissues (Lee and Rose, 2010; Xin and He, 2013). *Hpa* is an oomycete pathogen, also an agent of the downy mildew disease. As an obligate biotrophic pathogen, it keeps host cells alive for its growth and multiplication during all stages of infection (Coates and Beynon, 2010).

Many immune-related pathways, e.g., the salicylic acid and jasmonic acid/ethylene signaling pathways have been discovered through genetic or biochemical investigations of individual genes. Nevertheless, these investigations also indicate that a collection of interconnected pathways rather than individually unrelated genes are essential for plant resistance to pathogens (Wang L. et al., 2011; Ralhan et al., 2012). Therefore, it is necessary to understand plant–pathogen interactions from a systematic perspective (Elena and Rodrigo, 2012; Naseem et al., 2012; Weßling et al., 2014; Windram and Denby, 2015). A prominent example is the mapping of the protein–protein interaction (PPI) network between pathogen’s effectors and host proteins. In 2011, Mukhtar et al. (2011) constructed a plant–pathogen immune network (version 1; PPIN-1), which contains 3,148 interactions among 83 pathogen effectors from the aforementioned model pathogens *Psy* and *Hpa*, 170 immune proteins and 673 other *Arabidopsis* proteins. In this network, the NB-LRR immune receptors for ETI usually do not directly interact with the effectors, but tend to interact with host proteins targeted by the effectors (i.e., the effector targets). This observation supports the guard hypothesis in which immune proteins monitor effector targets to trigger ETI. This network also provides novel hypothesis and explanation regarding to the plant–pathogen interactions. For example, by analyzing real and random effector targets in PPIN-1, they found the effectors from *Psy* and *Hpa* are more likely to share targets compared with random, implying that these two diverse pathogens deploy effectors to a converged set of targets. They also observed higher degree (or connectivity) of real effector targets than random ones, indicating that the effectors tend to manipulate important host proteins in the network. This observation partly explains how the limited number of effectors could efficiently disrupt host immunity.

Although lots of progress has been made, there are still many questions that need to be further studied. What roles do the effector targets play in the network structure and organization? How are the targets of the same (or different) effector(s) distributed in the network? How does the network change dynamically in response to pathogen infection? Is there any underlying relationship between such dynamic changes and the effector target distribution? To this end, analyzing PPI network alone is apparently insufficient, and integrative network analyses are the excellent alternative choices. In fact, integrative network analyses have been successfully applied to the researches of host–pathogen interactions. For example, Gulbahce et al. (2012) focused on Epstein-Barr virus and human papillomavirus type 16, which are associated with Burkitt’s lymphoma and cervical cancer, respectively. They performed integrative network analysis to examine relative location between viral targets and disease susceptibility genes on the human interactome. The result shows that disease susceptibility genes are located in the network vicinity of viral targets. Recently, we combined machine learning method, modular network analysis and various types of data to investigate the shared and distinct network organization in *Arabidopsis* PTI and ETI (Dong et al., 2015). We found that the subnetwork shared by PTI and ETI is more likely to be targeted by pathogens. The previous hypothesis that the modular structures in ETI are relatively independent of each other for the robustness of ETI immunity, which was initially proposed based on the genetic associations between key immunity proteins (Tsuda et al., 2009), was also independently validated and extended to the interactome scale.

In this study, a comprehensive *Arabidopsis* PPI network was re-constructed and combined with known *Arabidopsis*-*Psy* and -*Hpa* PPIs. Firstly, we analyzed the topological features of the effector targets in the network, especially the targets interacting with the same effectors. Further, we integrated time series gene expression data to describe the dynamic network change during infection. Finally, a novel association between the dynamic network change and the effector target distribution was suggested, which also seemed to be predictive for the phenotypic outcomes (i.e., pathogen-susceptible or pathogen-resistant) of mutant plants.

## Results

### Assembly of a Comprehensive *Arabidopsis* PPI Network

We assembled a comprehensive *Arabidopsis* PPI network by merging experimental PPIs from different resources (BioGrid, IntAct, and TAIR databases, see Materials and Methods for details). The resulting network contained 23,797 PPIs among 8,519 proteins. Meanwhile, we collected *Arabidopsis-Psy* and *-Hpa* PPIs from a previous publication (Mukhtar et al., 2011) and mapped them onto the comprehensive *Arabidopsis* PPI network, in which the proteins interacting with the effectors were tagged as the effector targets. As a result, 52 *Arabidopsis* proteins (hereafter referred to as *Psy* targets) interacting with 27 *Psy* effectors and 109 *Arabidopsis* proteins (hereafter referred to as *Hpa* targets) interacting with 52 *Hpa* effectors were obtained, in which 17 *Arabidopsis* proteins were targeted by the effectors from both pathogens. Generally, each of the *Psy* and *Hpa* targets interacted with only one *Psy* effector and one *Hpa* effector, respectively (the median values are shown). On the contrary, each *Psy* or *Hpa* effector interacted with two *Arabidopsis* proteins (the median values are shown), which may be one of factors that enable pathogen infection through a handful of effectors (Li et al., 2012).

### Pathogens Employ the Local Impact Mode to Disrupt *Arabidopsis* Cellular Network

**FIGURE 1:**
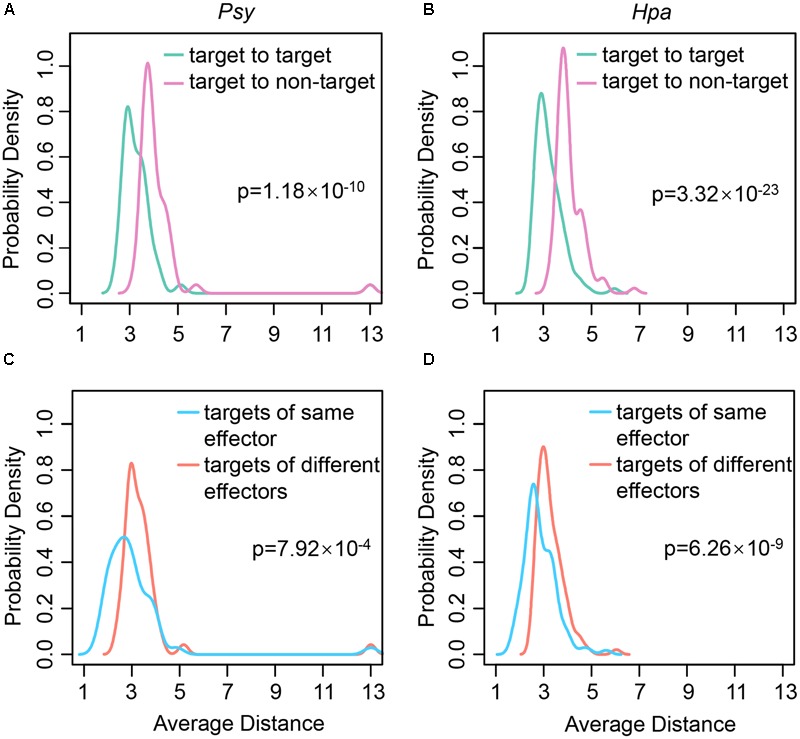
The distributions of average distances between different types of proteins. The distributions of average distances **(A)** from each of *Psy* targets to the rest of *Psy* targets and to non-*Psy* targets; **(B)** from each of *Hpa* targets to the rest of *Hpa* targets and to non-*Hpa* targets; **(C)** from each of *Psy* targets to the effector targets interacting with the same *Psy* effectors and to those interacting with different *Psy* effectors; **(D)** from each of *Hpa* targets to the effector targets interacting with the same *Hpa* effectors and to those interacting with different *Hpa* effectors in the comprehensive *Arabidopsis* PPI network are plotted. The significance of the difference in distance distributions is estimated using one-tailed Wilcoxon’s test.

One of the questions we would like to answer is what strategies pathogens employ to infect *Arabidopsis*. To this end, for each of *Psy* targets, we calculated the average distances from it to the other *Psy* targets and to the proteins not targeted by the *Psy* effectors (hereafter referred to as non-*Psy* targets; the non-*Hpa* targets were defined analogously). By comparison, we found that the average distances from *Psy* targets to *Psy* targets were significantly shorter than those from *Psy* targets to non-*Psy* targets (one-tailed Wilcoxon’s test, *p*-value = 1.18 × 10^-10^; **Figure 1A**), which indicates that *Psy* targets tend to converge onto local regions, rather than scatter across the whole network. Similarly, *Hpa* targets were also significantly closer to each other than non-*Hpa* targets (one-tailed Wilcoxon’s test, *p*-value = 3.32 × 10^-23^; **Figure 1B**). In other words, the *Hpa* effectors also tend to damage local regions in the comprehensive *Arabidopsis* PPI network (Supplementary Figure S1a). To be more straightforward, we identified 47 network modules by using the MCODE tool (Bader and Hogue, 2003), and found that the effector targets significantly clustered in five out of the 47 modules. The effector target distribution and the brief function annotation of these five modules are shown in Supplementary Figure S1b. In all, the results imply that the effectors tend to attack local regions in the host PPI network.

As described above, each effector can interact with multiple effector targets. We speculated that the effector targets interacting with the same effectors should also be located close to each other to confer potent local impact. To test this hypothesis, the distances between the targets of the same effectors and those between the targets of the different effectors were compared. Indeed, the distances between the targets of the same effectors were shorter, no matter for *Psy* targets (one-tailed Wilcoxon’s test, *p*-value = 7.92 × 10^-4^; **Figure 1C**) or for *Hpa* targets (one-tailed Wilcoxon’s test, *p*-value = 6.26 × 10^-9^; **Figure 1D**). It seems that each effector primarily disrupts a closely connected local region in the comprehensive *Arabidopsis* PPI network in order to quickly translocate the effector to all of its targets, resulting in an efficient local impact mode for interfering with the host cellular network.

We noticed that the above conclusion seems to conflict with previous result of Weßling et al. (2014). They constructed random networks by the degree-preserving rewiring method (Rao et al., 1996), and found that there were less direct interactions between effector targets in the real PPI network, in comparison with random networks (Weßling et al., 2014). Therefore, they concluded that effector targets were significantly dispersed than random expectation. To clarify this point, we also constructed 1,000 random networks based on the comprehensive *Arabidopsis* PPI network by using the same method. Indeed, the distances between different effector targets were significantly higher, on average, in the real network compared with those in the random networks (Supplementary Figures S2a–d). On the other hand, however, the distances between the effectors targets and non-targets were also significantly higher than random (Supplementary Figures S2e–h). In other words, the random network rewiring method reduced not only the target to target distances, but also the target to non-target distances. Therefore, the comparison of the target to target distances alone (Supplementary Figures S2a–d) would fail to accurately capture the relatedness between the target to target distances and target to non-target distances, as the changes in the target to non-target distances after random network rewiring (Supplementary Figures S2e–h) were omitted.

An alternative experiment to validate our observations is to check if the difference between target to target distances and target to non-target distances could be replicated in the random networks. More specifically, in each random network, we compared the distances between different effector targets and those between the effector targets and non-targets by one-tailed Wilcoxon’s test. If most random networks showed the *p*-values smaller than the *p*-values obtained from the real PPI network analysis, our observations could be randomly expected. Results demonstrated that most random networks did not show smaller *p*-values compared with the real network analysis: for *Psy*, 101 out of 1,000 random networks showed smaller *p*-values (Supplementary Figure S3a); while for *Hpa*, only three out of 1,000 random networks showed smaller *p*-values (Supplementary Figure S3b). Therefore, the observed smaller distances between different effector targets are not likely random. We further validated the observed smaller distances between the targets of the same effectors (**Figures 1C,D**) by the same experiment. The results indicate that this observation is also unlikely random. Only two out of 1,000 random networks showed smaller *p*-values for *Psy* (Supplementary Figure S3c), and none showed smaller *p*-values for *Hpa* (Supplementary Figure S3d).

In addition, the overall network topology would also influence our observations. To test this possibility, we introduced another frequently used *Arabidopsis* PPI network, termed AI-1_MAIN_ (Arabidopsis Interactome Mapping Consortium, 2011; Mukhtar et al., 2011; Weßling et al., 2014). This network was established based on the PPIs identified from one large scale yeast two-hybrid screen. Therfore, much less PPIs and proteins were included in AI-1_MAIN_ (5,664 PPIs among 2,661 proteins), compared with our comprehensive *Arabidopsis* PPI network (23,797 PPIs among 8,518 proteins). Nevertheless, 47 (90.38%) of *Psy* targets as well as 107 (98.17%) of *Hpa* targets were covered by AI-1_MAIN_, enabling this network as a choice for analyzing the influence of overall network topology. In this network, the distances between different effector targets, in comparison with those between the effector targets and non-targets, were significantly shorter (one-tailed Wilcoxon’s test, *p*-value = 5.59 × 10^-14^ for *Psy* and *p*-value = 1.58 × 10^-31^ for *Hpa*; Supplementary Figures S4a,b). Moreover, the targets of the same effectors were also significantly closer than those of the different effectors (one-tailed Wilcoxon’s test, *p*-value = 9.76 × 10^-4^ for *Psy* and *p*-value = 2.57 × 10^-8^ for *Hpa*; Supplementary Figures S4c,d). These results suggest the robustness of our observations to the alteration of overall network topology.

Finally, in addition to *Psy* and *Hpa* targets, a recent dataset of *Golovinomyces orontii* (*Gor*) targets is available (Weßling et al., 2014). *Gor* is an obligate biotrophic fungus and the pathogenic agent of powdery mildew on *Arabidopsis*. We validated our observations by using the *Gor* targets. Fifty *Arabidopsis* proteins (i.e., *Gor* targets) in the comprehensive *Arabidopsis* PPI network were targeted by 45 *Gor* effectors. Consistent with the results for *Psy* and *Hpa* targets, the *Gor* targets were also closer to each other in the comprehensive *Arabidopsis* PPI network (one-tailed Wilcoxon’s test, *p*-value = 8.99 × 10^-13^; Supplementary Figure S5a), so were the targets of the same *Gor* effectors (one-tailed Wilcoxon’s test, *p*-value = 0.013; Supplementary Figure S5b). Therefore, the effectors from different pathogens are likely to adopt similar local impact mode in order to efficiently attack host network.

### Key Positions Important for the Diffusion of Information throughout the Network Tend to be Targeted

**FIGURE 2:**
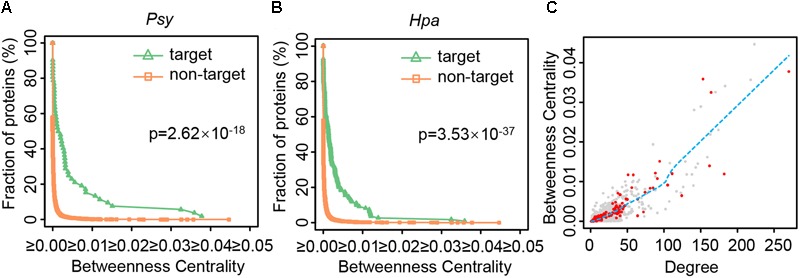
The distributions of betweenness centrality and degree for different types of proteins. **(A,B)** The cumulative distributions of the betweenness centrality of *Psy* targets, non-*Psy* targets, *Hpa* targets and non-*Hpa* targets in the comprehensive *Arabidopsis* PPI network are plotted after removing an outlier. The significance of the difference in betweenness centrality distributions is estimated using one-tailed Wilcoxon’s test. **(C)** The nodes represent all the proteins except for an outlier in the comprehensive *Arabidopsis* PPI network, of which red nodes represent both *Psy* targets and *Hpa* targets. The blue dashed line implies the tendency of betweenness centrality changing with degree, as estimated by the LOWESS smoothing method in R language.

How do local perturbations resulting from effector attacks propagate throughout the network, and finally cause the global change of the whole network? Intuitively, among the limited numbers of the effector targets, the proteins occupying the key positions in the network should be the first choices. Note that there are two types of key positions in the network. One is important for local network organization, characterized by higher degree. The other is important for global diffusion of information throughout the network, featured by higher betweenness centrality. It has been reported that host proteins targeted by pathogen proteins display higher degree (Li et al., 2012; Weßling et al., 2014; Halehalli and Nagarajaram, 2015; Memisevic et al., 2015), including those host proteins targeted by the *Psy* or *Hpa* effectors (Mukhtar et al., 2011). We also validated this tendency in our comprehensive PPI network (Supplementary Figure S6), indicating that the effector targets are indeed important for local network organization. However, such proteins are not always indispensable for global diffusion of information. As direct quantification, the betweenness centrality values of the effector targets and non-targets were compared. **Figure 2** depicts the cumulative distributions of the betweenness centrality of *Psy* targets and non-*Psy* targets, as well as those of *Hpa* targets and non-*Hpa* targets. For non-*Psy* or non-*Hpa* targets, the fraction of proteins decreased quickly as the betweenness centrality increases (**Figures 2A,B**), indicating only a few non-targets have high betweenness centrality. By contrast, the effector targets showed a significant shift toward higher betweenness centrality (one-tailed Wilcoxon’s test, *p*-value = 2.62 × 10^-18^ for *Psy* and *p*-value = 3.53 × 10^-37^ for *Hpa*). A protein with high betweenness centrality signifies that most information flows between the proteins in the network should pass this protein, and pathogens could disturb such proteins to paralyze global information diffusion of the host network.

Due to the specific structure of biological networks, a protein with higher degree naturally tends to have higher betweenness centrality. Indeed, the degree and betweenness centrality are highly correlated in the comprehensive *Arabidopsis* PPI network (**Figure 2C**; Spearman’s rank correlation coefficient = 0.889). To test the influence of degree or network topology on the above results, additional analyses were performed from the following three aspects. (a) We noticed that among proteins having higher degree (hubs), those targeted by effectors showed generally higher betweenness centrality (**Figure 2C**). We defined the proteins whose degrees were more than 5, 10, 15, or 20 as hubs in the comprehensive *Arabidopsis* PPI network, and re-performed the same analyses by considering the hubs only. Despite of different definitions of hubs, hubs targeted by effectors had higher betweenness centrality than other hubs (Supplementary Table S1). (b) We generated 1,000 random networks by the aforementioned degree-preserving rewiring method. For each network, we compared the betweenness centrality of the effector targets and non-targets, and quantified the significance of the difference by one-tailed Wilcoxon’s test. If most of random networks showed more significant difference (i.e., smaller *p*-values) than real PPI network, namely the comprehensive *Arabidopsis* PPI network, the result could be randomly expected. Out of 1,000 random networks, 325 and four showed smaller *p*-values, for *Psy* and *Hpa*, respectively (Supplementary Figure S7). It is noteworthy that the random networks were constructed by the degree-preserving rewiring method where the degree of each protein was fully kept, so did the strong correlation between degree and betweenness centrality (average Spearman’s rank correlation coefficient = 0.944). Under this configuration, it should be hard to alter one protein’s betweenness centrality when its degree remains unchanged. Nevertheless, less than one third of random networks could replicate the result from the real network (Supplementary Figure S7), indicating that the higher betweenness centrality of effector targets, though strongly influenced by degree, is not likely random. (c) We examined the effector targets and non-targets’ betweenness centrality in AI-1_MAIN_. Similar to the result from the comprehensive *Arabidopsis* PPI network, the significantly higher betweenness centrality of the effector targets was observed (one-tailed Wilcoxon’s test, *p*-value = 1.11 × 10^-15^ for *Psy* and *p*-value = 4.54 × 10^-34^ for *Hpa*; Supplementary Figure S8). These results together indicate that, though the betweenness centrality is strongly influenced by degree, the effector targets’ higher betweenness centrality is not likely to simply result from their higher degree, but could be considered as another aspect of the topological feature of the effector targets. In addition, by evaluating the difference in the betweenness centrality distributions of the *Gor* targets and non-*Gor* targets in the comprehensive *Arabidopsis* PPI network, we found the *Gor* targets also had significantly higher betweenness centrality (one-tailed Wilcoxon’s test, *p*-value = 1.71 × 10^-20^; Supplementary Figure S9), suggesting the potential generalizability of this finding.

To further simulate the impact of effector attack on the diffusion of information in the comprehensive *Arabidopsis* PPI network, we first employed the time series gene expression datasets of *Arabidopsis* inoculated with *Psy* (GSE5685) or *Hpa* (GSE22274) to assess the dynamic network change during infection. The first dataset (GSE5685) produced by the AtGenExpress project contains five different time points: 4, 8, 16, 24, and 48 h post-inoculation (hpi). The second dataset (GSE22274) was generated by Wang W. et al. (2011) and detected at four different time points: 0.5, 2, 4, and 6 days post-inoculation (dpi). The up-regulated DEGs (activated genes) during *Psy* or *Hpa* infection were identified at each time point and mapped onto the comprehensive *Arabidopsis* PPI network (Supplementary Table S2 and Figure S10). In total, 465 up-regulated DEGs (hereafter referred to as *Psy* DEGs) and 614 up-regulated DEGs (hereafter referred to as *Hpa* DEGs) in response to *Psy* and *Hpa* infections were obtained at different time points, respectively. We computed the average distances between DEGs of any two adjacent time points in the comprehensive *Arabidopsis* PPI network for *Psy* and *Hpa* as the measurement of baseline efficiency of network information diffusion (**Table 1**). Subsequently, *Psy* or *Hpa* targets were removed from the comprehensive *Arabidopsis* PPI network. Here, we assumed that the DEGs were not changed with the effector target removal because it was very hard to know what DEGs were actually changed. With this assumption, we instead focused on the changes of the relationship between DEGs. More specifically, if the average distances between DEGs of two adjacent time points become higher after the removal of the effector targets, the declining efficiency of network information diffusion would be assumed. Indeed, the removal of either *Psy* or *Hpa* targets could increase the distances between DEGs of adjacent time points (**Table 1**), thus likely disturbing the diffusion of information.

**Table 1:** The comparison of the average distances between DEGs of two adjacent time points before and after removing the effector targets from the comprehensive *Arabidopsis* PPI network.

	*Psy* infection				*Hpa* infection		
Time^a^	4 hpi- > 8 hpi	8 hpi- > 16 hpi	26 hpi- > 24 hpi	24 hpi- > 48 hpi	0.5 dpi- > 2 dpi	2 dpi- > 4 dpi	4 dpi- > 6 dpi
+ Targets^b^	2.467	2.052	1.114	1.319	1.692	1.518	1.096
- Targets^c^	2.717	2.273	1.460	1.472	1.949	1.967	1.272

*^a^Two adjacent time points during *Psy* or *Hpa* infection. ^b^The average distances between DEGs of two adjacent time points when effector targets are included in the comprehensive *Arabidopsis* PPI network. ^c^The average distances between DEGs of two adjacent time points after removing *Psy* or *Hpa* targets from the comprehensive *Arabidopsis* PPI network.*

Similarly, we mapped the up-regulated DEGs during *Psy* or *Hpa* infection onto AI-1_MAIN_ (Supplementary Table S3) and found that the results remain unchanged, i.e., after removing the effector targets, the distances between DEGs of adjacent time points also increase (Supplementary Table S4). We also tried to perform the same analyses for *Gor* infection. Unfortunately, however, there were not sufficient DEGs identified from the time series gene expression dataset with respect to *Gor* infection (GSE13793). For example, only one DEG could be identified at 3 dpi. Therefore, we did not perform subsequent DEG-centered analyses for *Gor*.

### Increasing Distances from DEGs to the Effector Targets during Infection

In the above analyses, we have shown that the distribution of the effector targets in the network is non-random. Intuitively, the *Arabidopsis* network should have specific changes in response to such non-random attack pattern. We first examined the expression change of *Psy* and *Hpa* targets during infection. Only four (7.69%) out of *Psy* targets and six (5.50%) out of *Hpa* targets were up-regulated during infection, indicating that the effector targets themselves are not likely to be activated during infection. Nevertheless, we found that most of DEGs were located in the vicinity of the effector targets in the network (Supplementary Figure S11). For 436 (93.76%) out of 465 *Psy* DEGs, their distances to *Psy* targets were no more than three (Supplementary Figure S11a). Likewise, for 587 (95.60%) out of 614 *Hpa* DEGs, their distances to *Hpa* targets were no more than three (Supplementary Figure S11b). This result indicates that *Arabidopsis* mostly activates the genes in the vicinity of the effector targets in the network for defense response.

**FIGURE 3:**
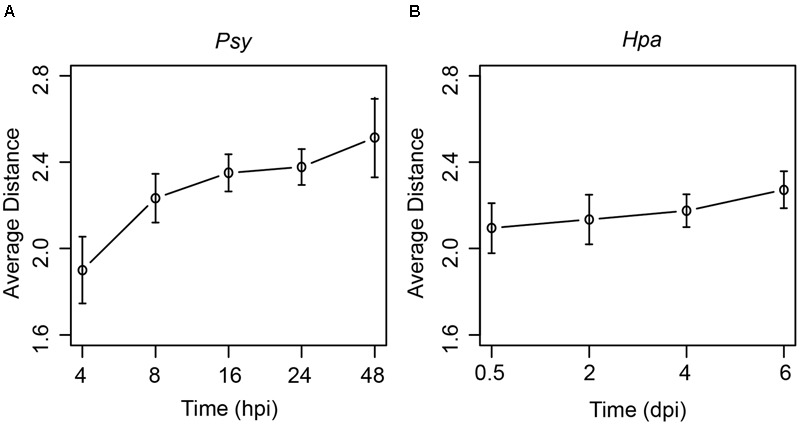
The changes of the average distances from DEGs at different time points to the effector targets. The average distances from DEGs at each time points during **(A)** *Psy* or **(B)** *Hpa* infection to *Psy* or *Hpa* targets in the comprehensive *Arabidopsis* PPI network are plotted. Error bars represent the standard errors of the average distances.

On the other hand, *Arabidopsis* response to pathogen invasion is a dynamic process, and genes with specific functions would be activated at specific stages of the infection (Lewis et al., 2015). To explore dynamic network changes, at each time point during *Psy* or *Hpa* infection, we computed the average distances between DEGs and the effector targets (see Materials and Methods). Interestingly, both *Psy* and *Hpa* DEGs followed the same trend: DEGs at the first time point (4 hpi for *Psy* and 0.5 dpi for *Hpa*) were located closest to the effector targets; and the average distances between DEGs and the effector targets increased over time (**Figure 3**). To evaluate the significance of this result, for DEGs at each time point, we randomly picked equal number of proteins from the comprehensive *Arabidopsis* PPI network and calculated their distances to the effector targets. Such random trial was repeated 1,000 times. Subsequently, we counted the number of trials where consistently increasing distances from randomly picked proteins to the effector targets could be observed. Only 22 and 36 out of 1,000 random trials showed consistently increasing distances over all time points, for *Psy* and *Hpa*, respectively (Supplementary Figure S12). We also tried to relax the restriction by counting the number of trials where consistently increasing distances over the first four, first three or first two time points for *Psy*. As expected, consistently increasing distances over at least three time points were unlikely observed in random trials (Supplementary Figure S12a). Similarly, we could observe only 139 out of 1,000 random trials where the distances increased over the first three time points (Supplementary Figure S12b). These results indicate that consistently increasing distances to the effector targets could imply a non-random regulation of gene expression in response to pathogen infection.

We checked the robustness of this observation to the changes of network topology. Firstly, a larger *Arabidopsis* network, which was constructed by combining the confirmed protein-DNA interactions from the AGRIS database (Yilmaz et al., 2011), and known *Arabidopsis* PPIs were employed. The resulting network consists of 23,797 PPIs and 4,217 protein-DNA interactions. Similar to the results obtained from the comprehensive *Arabidopsis* PPI network, the average distances between DEGs and the effector targets increased over time in this larger network (Supplementary Figure S13). Secondly, we also validated the observation by randomly removing 10, 20, 30, and 40% PPIs, or 10, 20, 30, and 40% effector targets from the comprehensive *Arabidopsis* PPI network. Again, the generally similar trends were observed after the removal of PPIs (Supplementary Figure S14) or effector targets (Supplementary Figure S15). We also noticed that the increasing trends at later time points for *Psy* could be slightly disrupted with the PPI removal (Supplementary Figure S14). Nevertheless, the general increasing trend for the distances from DEGs to the effector targets, especially at the early time points, remained stable. Finally, we performed the analysis in AI-1_MAIN_. For *Hpa*, consistently increasing distances were validated (Supplementary Figure S16b). But for *Psy*, the increasing distances were observed only at the early time points, and the increasing trend was disrupted at the later time points (Supplementary Figure S16a), mimicking the results after the PPI removal. In fact, about 76.2% of PPIs in the comprehensive *Arabidopsis* PPI network were removed in AI-1_MAIN_. Therefore, this disruption of the increasing trend was plausibly due to the limited size of AI-1_MAIN_, since many connections between DEGs and the effector targets were not included in AI-1_MAIN_.

In summary, a specific dynamic trend of DEGs in relation to the effector targets has been observed, in which the DEGs seemed to step away from the effector targets during infection. One may notice that only up-regulated DEGs were considered here. This is because most DEGs were up-regulated (activated) during infection. Indeed, analyzing both up-regulated and down-regulated DEGs would result in the trends highly similar to those observed for up-regulated DEGs (Supplementary Figure S17a), even though down-regulated DEGs showed somewhat different trends (Supplementary Figure S17b).

### Pathogen-Susceptible Mutants Tend to have More DEGs near Effector Targets than Pathogen-Resistant Ones

We speculated that the aforementioned dynamic network pattern, where DEGs exhibit increasing distances to effector targets, has its merits in the immune response. That is to say, DEGs should not stay in the region adjacent to the effector targets, otherwise the plant could be susceptible to infection. To test this possibility, we compared the average distances between the effector targets and the DEGs in pathogen-susceptible mutants, with average distances between the effector targets and the DEGs in pathogen-resistant mutants. Expression profiles of eight *Arabidopsis* mutants and the corresponding wild-types were collected after inoculation with *Psy* (**Table 2**). Of these mutated genes, the products of *PHYTOALEXIN DEFICIENT 4 (PAD4), PATHOGENESISRELATED GENES 1 (NPR1)* and *SA INDUCTION DEFICIENT 2* (*SID2)* are indispensable members of SA signaling pathway. Their mutants are more susceptible to *Psy* in comparison to wild-type, so is the *phytoalexin deficient 2 (pad2)* mutant (Vlot et al., 2009; Dubreuil-Maurizi et al., 2011; Shearer et al., 2012; Tsuda et al., 2013). While for *CORONATINE INSENSITIVE 1 (COI1)* and *ETHYLENE INSENSITIVE 2 (EIN2)* whose products are important constituents of JA-ET signaling pathways, enhanced resistance is displayed in their mutants during *Psy* infection. Likewise, *lysine histidine transporter 1 (lht1)* and *wrky18/40* mutants also display enhanced resistance to *Psy* (Wang et al., 2002; Eulgem and Somssich, 2007; Liu et al., 2010; Ralhan et al., 2012; Zheng et al., 2012; Cho and Yoo, 2014). We also obtained expression profiles of four *Arabidopsis* mutants and the corresponding wild-types after inoculation with *Hpa* (**Table 2**). In *wrky72* mutant and *recognition of peronospora parasitica 4 (rpp4)* mutant, more susceptible phenotypes are conferred in response to *Hpa* infection (Bhattarai et al., 2010; Wang W. et al., 2011). In contrast, *microtubule-associated protein 65-3 (map65-3)* and *phytosulfokin receptor 1 (pskr1)* mutants show enhanced disease tolerance to *Hpa* (Quentin et al., 2016; Rodiuc et al., 2016).

**Table 2:** The average distances from the DEGs in susceptible or resistant mutants to the effector targets.

Series	Platform	Mutated gene^a^	Phenotype	#DEG^b^	Distance^c^	Rank^d^
***Psy* infection**				
GSE6829	GPL198	*wrky18/40*	Resistant	242	2.292	1
GSE18978	GPL198	*ein2*	Resistant	225	2.249	2
GSE18978	GPL198	*coi1*	Resistant	621	2.214	3
GSE19109	GPL198	*lht1*	Resistant	696	2.206	4
GSE18978	GPL198	*pad4*	Susceptible	1025	2.205	5
GSE18978	GPL198	*npr1*	Susceptible	602	2.198	6
GSE18978	GPL198	*pad2*	Susceptible	234	2.176	7
GSE18978	GPL198	*sid2*	Susceptible	346	2.175	8
***Hpa* infection**				
GSE73351	GPL198	*map65-3*	Resistant	50	2.122	1
GSE37255	GPL198	*pskr1*	Resistant	29	2.036	2
GSE18329	GPL198	*wrky72*	Susceptible	109	2.000	3
GSE22274	GPL198	*rpp4*	Susceptible	50	2.000	4

*^a^Mutants are more resistant or susceptible (column 4) to *Psy* or *Hpa* compared with wild-types. ^b^The number of genes differentially expressed in mutants (column 3) versus the corresponding wild-types, after inoculation with *Psy* or *Hpa*. ^c^The average distances between the DEGs and *Psy* or *Hpa* targets in the comprehensive *Arabidopsis* PPI network. ^d^The distances (column 6) are ranked in descending order.*

**FIGURE 4:**
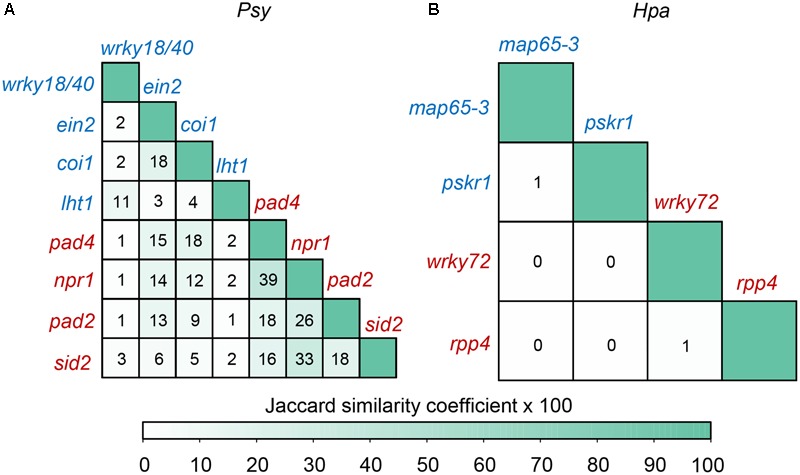
The similarities between DEGs in different mutants. The DEGs are obtained by comparing mutants versus wild-type, after *Psy* or *Hpa* infection. The similarity between two DEG sets in different mutants in the context of **(A)** *Psy* or **(B)** *Hpa* infection is estimated by Jaccard similarity coefficient. The Jaccard similarity coefficient is calculated by taking the number of DEGs involved in both of the two sets divided by the number of DEGs involved in either of the two sets. The value marked in the cell is the product of the Jaccard similarity coefficient and 100.

We identified up-regulated DEGs upon each inoculated mutant in comparison with inoculated wild-type, and measured the average distance from each set of DEGs to the corresponding *Psy* or *Hpa* targets in the comprehensive *Arabidopsis* PPI network (**Table 2**). Intriguingly, the mutant phenotypes can be largely separated based on the distances. DEGs in *Psy* or *Hpa*-susceptible mutants were closer to *Psy* or *Hpa* targets, respectively, relative to DEGs in *Psy* or *Hpa*-resistant mutant (**Table 2**). These results indicate that too close allocation of DEGs to the effector targets is correlated with pathogen susceptibility. Currently, we cannot distinguish which is the effect and which is the cause from this observed correlation. Nevertheless, this correlation is at least not likely to be a simple result of the similarity between DEGs, because the similarities between DEGs in different mutants were generally limited: none of the pair-wise Jaccard similarity coefficients between two sets of mutant DEGs was higher than 0.4, even restricting the same phenotypes (**Figure 4A**). More prominently, the Jaccard similarity coefficients, except for those between DEGs in *rpp4* and *wrky72* mutants and between DEGs in *pskr1* and *map65-3* mutants, were 0 in the context of *Hpa* infection (**Figure 4B**), thus it is difficult to infer phenotypes simply by the similarity between DEGs. We validated the above observation by randomly removing 10, 20, 30, or 40% PPIs from the comprehensive *Arabidopsis* PPI network and re-calculated the average distances from each set of DEGs to the corresponding *Psy* or *Hpa* targets after the removal of PPIs. Generally, DEGs in *Psy* or *Hpa*-susceptible mutants were closer to *Psy* or *Hpa* targets compared with DEGs in *Psy* or *Hpa*-resistant mutants, though the rank was not perfectly in line with the phenotypic discrimination (*sid2* was misclassified, Supplementary Table S5). We then tested our finding in AI-1_MAIN_, where near 80% of PPIs have been removed. For *Psy*, one mutant was misclassified (*coi1* was misclassified, Supplementary Table S6), mimicking the above result after PPI removal. But for *Hpa*, only a handful of DEGs (19 DEGs on average) were covered by AI-1_MAIN_, and the ranks of distances failed to accurately discriminate different phenotypic outcomes (Supplementary Table S6). This result indicates that sufficient coverage of PPIs (and DEGs) is required to observe the correlation between the DEG to effector target distance and phenotypic outcome.

We also employed another sets of DEGs where the expression profiles of *Arabidopsis* mutants before and after inoculation were compared instead. More specifically, the genes up-regulated in *Arabidopsis* mutants after *Psy* or *Hpa* inoculation were treated as the DEGs. Consistent with the above results, the distances from DEGs to the effector targets in the comprehensive *Arabidopsis* PPI network had the ability to distinguish phenotypes (Supplementary Table S7), while the similarity between DEGs even for mutants of the same phenotype was consistently low (Supplementary Figure S18). Undoubtedly, the low reproducibility between the different experiments should be one reason of the low similarity. However, even for the mutants tested in the same transcriptome profiling experiment, such as *med16, med 14* and *npr1* (GSE45214; Supplementary Table S7), the overlapped DEGs were still limited (Supplementary Figure S18). Unlike NPR1, MED14 and MED16 are not only involved in salicylic acid signaling pathway but also involved in jasmonic acid/ethylene signaling pathway (Shearer et al., 2012; Wang et al., 2016). Moreover, it has been reported that MED14 and MED16 differentially regulate defense gene expression in plant immune responses (Zhang et al., 2013). Therefore, we speculate that the activated pathways in the three mutants could be different, which may be another factor that gives rise to the low similarity of DEGs. In a word, above results indicate that the distance from DEGs to the effector targets could be a network parameter of biological significance which is associated with the phenotypic outcomes.

## Discussion

Plant–pathogen interactions are complex and dynamic. In general, pathogens including bacteria, oomycetes and fungi secrete effectors to manipulate plant proteins with important physiology functions (Mukhtar et al., 2011; Weßling et al., 2014). To defense pathogens, plants have evolved a series of sophisticated immune mechanisms (van Schie and Takken, 2014). In this study, we obtained the valuable dataset of effector-target interactions from Mukhtar et al. (2011) and Weßling et al. (2014), which is an essential knowledge to explore the principles of pathogenicity and plant immunity. Further, we performed more extensive and meticulous analyses by integrating other large-scale datasets. In summary, there are three advantages in our study. Firstly, by analyzing a comprehensive *Arabidopsis* PPI network, we have found that the effectors locally and densely disrupt proteins important for the information diffusion throughout the network. This observation partly explains the efficiency of pathogen infection strategies from the network topology aspects. Secondly, by further integrating time series gene expression data, an interesting pattern of the changes in the distances between the DEGs and the effector targets has been revealed. Last but not least, by comparing the DEGs in susceptible and resistant mutants, we have found that the distances between the DEGs and the effector targets also have a phenotype implication.

### The Complexity of Effector-Target Interaction Topology

The interactions between the effectors and the targets are promiscuous. A single *Psy* or *Hpa* effector can interact with multiple *Arabidopsis* proteins and even disorganize diverse biological processes, which may explain why about 30 *Psy* effectors or about 130 *Hpa* effectors can fight with *Arabidopsis* containing nearly 30,000 protein coding genes (Coates and Beynon, 2010; Xin and He, 2013; Lewis et al., 2014). *Psy* effector AvrPto is a classic example. It directly binds 11 *Arabidopsis* proteins and can manipulate immune receptor kinases to subvert plant immune system (Win et al., 2012). ATR13 is a *Hpa* effector, and it interacts with 24 *Arabidopsis* proteins that involve in different biological processes including regulation of transcription, metabolism, and nucleotide biosynthetic process based on GO annotations by the DAVID tool (Huang da et al., 2009). Our results have showed that the effector targets tend to have higher degree and betweenness centrality. Considering the wide spectrum of biological functions of the effector targets, it is possible that some effector targets play an important role in linking multiple gene modules with different biological functions.

### The Relationships among DEGs, Effector Targets, and Phenotypes

As observed in our analysis, DEGs at the first time point are closest to the effector targets. And the distances from DEGs to effector targets seemed to be gradually increased over time. Such specific change in the relative locations between the effector targets and DEGs at different time points can be explained from two perspectives that are not mutually exclusive. On the one hand, DEGs closest to the targets in the comprehensive *Arabidopsis* PPI network are activated at the soonest, followed by spread the information to other DEGs step by step, triggering the immune response. On the other hand, the long-term evolutionary arms race between *Arabidopsis* and the pathogens urges DEGs that involve in the plant immunity to step away from the effector targets for eluding the adverse impact from effectors’ attack. In line with this assumption, the mutants whose DEGs are located farther from the effector targets are more likely associated with resistant phenotype. On the contrary, the mutants whose DEGs are located closer to the effector targets are more likely associated with susceptible phenotype. Albeit the above intuitive explanations and preliminary validations, the generalizability and mechanism underlying this network topological pattern between DEGs and the effector targets needs further extensive experimental validations and investigations.

### Limitation and Future Work

To date, experimental plant–pathogen PPIs including *Arabidopsis*-*Psy* and -*Hpa* PPIs, and time series gene expression data after inoculation with pathogens are rare, mainly because the detection technology is challenging and time-consuming (Nourani et al., 2015). In addition, it was estimated that current experimental PPIs covers only a small proportion of plant interactomes (Gu et al., 2011). As a result, low coverage of plant PPIs, incomplete plant–pathogen interactions or a handful of time series datasets may lead to biased results.

Nevertheless, our preliminary results have provided interesting clues for further analysis. By comparing the effector targets and non-targets in this study, we have found that the effector targets possess unique characteristics, which can be considered as features in computational methods to predict effector-target interactions. Further, based on experimental and high-quality predicted datasets, our analysis may be extended to other plant species like rice and maize to test if our findings could be widely consolidated. Moreover, the feature of the distance between DEGs and the effector targets may contribute to the prediction of genes related to plant resistance or susceptibility.

Finally, it is expected that complementary data, when available, would further deepen our understanding of plant–pathogen interaction. The integration of pathogens’ transcriptome data, especially time series gene expression data, is an effective measure (Westermann et al., 2012). On the one hand, integrating the time series datasets of pathogens could aid the prediction of the sequential order of effector secretion *in planta*, so that the importance of the effector combinations could be evaluated. On the other hand, by integrating dual transcriptome data from both the pathogen and the host, one can analyze the associations between changes in the transcriptomes of pathogens and plants, and thus better understand pathogenicity and plant immunity (Westermann et al., 2012). In addition, three-dimensional protein structures of effectors and targets could also be helpful. By identifying the key residues for effector-target interactions in the three-dimensional protein structures, we can modify the residues of effector targets to prevent detrimental plant–pathogen interactions, or creates decoys for effectors to monitor pathogen invasion and trigger plant immunity (Nishimura et al., 2015).

## Conclusion

We have conducted an integrative network analysis by combining *Arabidopsis* PPIs, known targets of pathogen effectors and *Arabidopsis* gene expression profiles in the immune response. The results show that, despite different mechanisms to colonize plants and different lifestyles in plant cells, the strategies for host network attack by *Psy* and *Hpa* are surprisingly similar. For both pathogens, the effector targets are closer to each other in the comprehensive *Arabidopsis* PPI network compared with non-targets, in particular, the targets that interact with the same effectors are closer to each other compared with the targets that interact with different effectors. These results imply that the pathogens employ the local impact mode for network attack where each effector damages a tightly connected region in the *Arabidopsis* PPI network. In comparison to non-targets, the effector targets have higher betweenness centrality, indicating the proteins important for the information diffusion throughout the network are the first choice of the effector targets. This observation also partly explains why the pathogens’ local impact mode could have global influences upon the host cellular network.

In response to the non-random distribution of the effector targets in the network, the specific allocation pattern of *Arabidopsis* DEGs is revealed. As observed when analyzing two distinct time-series transcriptome data, DEGs as first responders are closest to the effector targets and the distances between DEGs and the effector targets increase over time. Detailed analyses further reveal that DEGs in susceptible mutants are closer to the effector targets compared with those in resistant mutants.

Collectively, our analyses suggest a common topological relationship between DEGs and the effector targets in the network. While pathogens employ potent local impact mode to interfere with key positions in host network, plant organizes an in-depth defense by sequentially activating genes distal to the effector targets. The feature analyses of the effector targets will facilitate the discovery of potential targets, while the analyses of the distances between DEGs and the effector targets may provide novel clues for the identification of the genes conferring pathogen susceptibility or resistance.

## Materials and Methods

### Collecting *Arabidopsis* and *Arabidopsis*-Pathogen PPIs

*Arabidopsis* binary PPIs were downloaded from the BioGRID (Chatr-Aryamontri et al., 2015), IntAct (Orchard et al., 2014) and TAIR (Lamesch et al., 2012) databases in July, 2015. To merge the PPIs from different interaction repositories, the identifier of each protein was remapped to the TAIR identifier using the ID mapping tool in the UniProt database (UniProt Consortium, 2015). As a result, a comprehensive *Arabidopsis* PPI network including 23,797 non-redundant *Arabidopsis* PPIs among 8,519 proteins was constructed after discarding unmapped PPIs and self-interactions. More, collapsed *Arabidopsis*-*Psy* and -*Hpa* PPI data, in which PPIs containing the effectors from the same loci were merged, were retrieved from the previous publication (Mukhtar et al., 2011). Finally, 96 interactions between 27 *Psy* effectors and 52 *Arabidopsis* targets (i.e., *Psy* targets), and 220 interactions between 52 *Hpa* effectors and 109 *Arabidopsis* targets (i.e., *Hpa* targets) were obtained. By the degree-preserving rewiring method in the R package igraph (Csardi and Nepusz, 2006), 1,000 random networks were constructed. Just as its name implies, the degree-preserving rewiring method randomly rewires the real network’s edges while preserving the degrees of all nodes. Technically, this procedure could be achieved by choosing two arbitrary edges A-B and C-D, and substituting them with A-D and C-B. We also employed AI-1_MAIN_, a network derived from one large scale yeast two-hybrid assay (Arabidopsis Interactome Mapping Consortium, 2011; Mukhtar et al., 2011; Weßling et al., 2014). AI-1_MAIN_ included 2,661 proteins and 5,664 PPIs (after excluding self-interactions). To test the generalizability of topology features observed for *Psy* and *Hpa* targets, the *Gor* targets derived from Weßling et al. (2014), if applicable, were also introduced in the analyses.

### Calculating the Distances between Proteins

By definition, the distance between two nodes (proteins) in the network was equal to the length of the shortest path between them. Note that the network diameter was assigned as the distance if two nodes were from two separated (i.e., not mutually connected) parts of the network. We employed the igraph package (Csardi and Nepusz, 2006) in R language^1^ to calculate the distance between two nodes. The calculation for the average distance between a differentially expressed gene (DEG) set and an effector target set was performed in two steps. Firstly, the minimum distance between a DEG to any effector target in the effector target set was calculated as the distance from this DEG to the effector target set. Secondly, the distances between any DEG in the DEG set to the effector target set were averaged. The average distance between the DEG sets of two adjacent time points was calculated in a similar fashion, where the DEG set of the previous time point and that of the next time point were analogous to the effector target set and the DEG set, respectively. When plotting the distributions of distances, the Gaussian kernel with default parameters, implemented by the ’density’ function in the ‘stats’ package of R (version 3.1.0), was employed to smooth the distributions of average distances between different types of proteins.

### Calculating the Topological Parameters of Proteins

The degree and betweenness centrality of each protein in networks were measured using the igraph package (Csardi and Nepusz, 2006) in R language^1^. The degree of a protein denotes the number of partners in networks, while the betweenness centrality of a protein measures the number of shortest paths from all proteins to all others passing through this protein. The higher betweenness centrality of a protein means that more shortest paths should pass through this protein in networks. Usually, the proteins important for the information propagation throughout networks have higher betweenness centrality.

### Processing Gene Expression Data and Defining DEGs

Firstly, the time series gene expression datasets during *Psy* and *Hpa* infections (GSE5685 and GSE22274), and the comparative gene expression profiles of *Arabidopsis* mutants in response to *Psy* or *Hpa* inoculation were collected from the Gene Expression Omnibus (GEO) database (Barrett et al., 2013). Next, raw expression data were normalized utilizing the RMA method provided by the Bioconductor affy package (Gautier et al., 2004) in R language. Meanwhile, probe IDs were remapped to TAIR identifiers. Further, we defined up-regulated DEGs which satisfied both the fold change (at least 1.5) and *p*-value (less than 0.05 after *t*-test) criteria simultaneously. It has been shown that the combination of the two criteria contributes to better microarray interpretations (McCarthy and Smyth, 2009; Dalman et al., 2012). For the time series datasets, the fold change was acquired upon pathogen inoculation treatment as compared to control. For the comparative gene expression profiles, two types of DEGs were obtained by comparing the gene expression of (a) pathogen-inoculated mutant vs. pathogen-inoculated wild-type and (b) pathogen-inoculated mutant vs. non-inoculated mutant, respectively. Note that only the up-regulated DEGs appearing in the comprehensive *Arabidopsis* PPI network were included in the analyses of this study.

### Quantifying the Similarity between Two DEG Sets

Jaccard similarity coefficient was applied to evaluate the similarity between two DEG sets. It was calculated by taking the number of DEGs involved in both sets (i.e., the intersection of two sets) divided by the number of DEGs involved in either of the two sets (i.e., the union of two sets). The higher Jaccard similarity coefficient is, the more similar two DEG sets are.

## Author Contributions

HL designed the study, performed the analyses and drafted the manuscript; YZ and ZZ initiated, designed and supervised the study, and revised the manuscript.

## Conflict of Interest Statement

The authors declare that the research was conducted in the absence of any commercial or financial relationships that could be construed as a potential conflict of interest.

## Footnotes

1 https://www.r-project.org/
