# Supplementary material for: Network Analysis Reveals a Common Host–Pathogen Interaction Pattern in Arabidopsis Immune Responses
Source: Front Plant Sci. 2017 May 29;8:893. doi: 10.3389/fpls.2017.00893 (PMC5446985; doi:10.3389/fpls.2017.00893)
Supplement: Supplementary file 1 [file Presentation_1.pdf]

# Supplementary Figures and Tables

## Network analysis reveals a common host-pathogen interaction pattern in Arabidopsis immune responses

Hong Li, Yuan Zhou* and Ziding Zhang*

***Corresponding authors**: Yuan Zhou (soontide6825@163.com), Ziding Zhang (zidingzhang@cau.edu.cn)

**Table of contents:**

**Figure S1** The distribution of the effector targets in the comprehensive *Arabidopsis* PPI network.

**Figure S2** The comparison of the distances between different types of proteins, between real and random networks.

**Figure S3** The p-values for the comparison of the distances between different types of proteins, in the real PPI network and random networks, respectively.

**Figure S4** The distributions of average distances between different types of proteins in AI-$1_{MAIN}$.

**Figure S5** The distributions of average distances between different types of proteins for *Gor* in the comprehensive *Arabidopsis* PPI network.

**Figure S6** The cumulative distributions of the degree of the effector targets and non-targets in the comprehensive *Arabidopsis* PPI network.

**Figure S7** The p-values for the comparison of betweenness centrality, in the real PPI network and random networks, respectively.

**Figure S8** The cumulative distributions of the betweenness centrality of the effector targets and non-targets in AI-$1_{MAIN}$.

**Figure S9** The cumulative distributions of the betweenness centrality of the *Gor* targets and non-*Gor* targets in the comprehensive *Arabidopsis* PPI network.

**Figure S10** Venn-diagrams depicting the number of overlapped DEGs between different time points.

**Figure S11** The minimum distances from each of *Psy* (or *Hpa*) DEGs to *Psy* (or *Hpa*) targets in the comprehensive *Arabidopsis* PPI network.

**Figure S12** The frequency for observing consistently increasing distances between random proteins and the effector targets in 1,000 random trials.

**Figure S13** The changes of the average distances from DEGs at different time points to the effector targets in a larger *Arabidopsis* network.

**Figure S14** The changes of the average distances from DEGs at different time points to the effector targets after removing different proportions of PPIs.

**Figure S15** The changes of the average distances from DEGs at different time points to the rest of effector targets after removing different proportions of effector targets.

**Figure S16** The changes of the average distances from DEGs at different time points to the effector targets in AI-$1_{MAIN}$.

**Figure S17** The changes of the average distances for different types of DEGs at different time points to the effector targets.

**Figure S18** The similarities between DEGs in different mutants using different definitions of DEGs.

**Table S1** The comparison of the betweenness centrality distributions between the effector targets and non-targets among hubs.

**Table S2** The number of DEGs at different time points during infection in the comprehensive *Arabidopsis* PPI network.

**Table S3** The number of DEGs at different time points during infection in AI-$1_{MAIN}$.

**Table S4** The comparison of the average distances between DEGs of two adjacent time points before and after removing the effector targets from AI-$1_{MAIN}$.

**Table S5** The average distances from the DEGs in susceptible or resistant mutants to the effector targets after removing different proportions of PPIs.

**Table S6** The average distances from the DEGs in susceptible or resistant mutants to the effector targets in AI-$1_{MAIN}$.

**Table S7** The average distances from the DEGs in susceptible or resistant mutants to the effector targets using different definitions of DEGs in the comprehensive *Arabidopsis* PPI network.

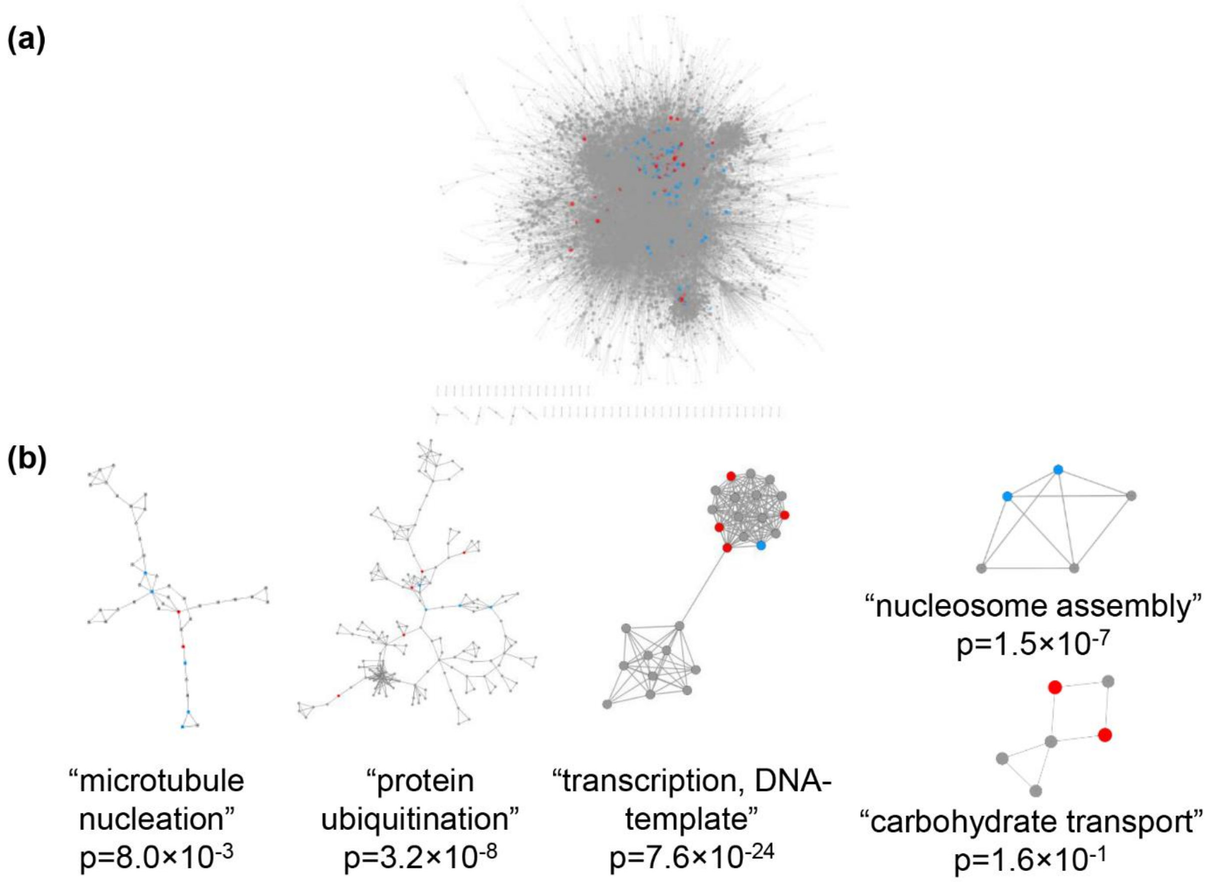


**Figure S1 The distribution of the effector targets in the comprehensive *Arabidopsis* PPI network.**

*Psy* and *Hpa* targets are highlighted using red and blue. (a) The overall distribution of the effector targets in the comprehensive *Arabidopsis* PPI network. (b) The five target-enriched modules. For each module, its most significantly associated GO term and the corresponding p-value (Fisher’s exact test followed by Benjamini-Hochberg correction) are shown.

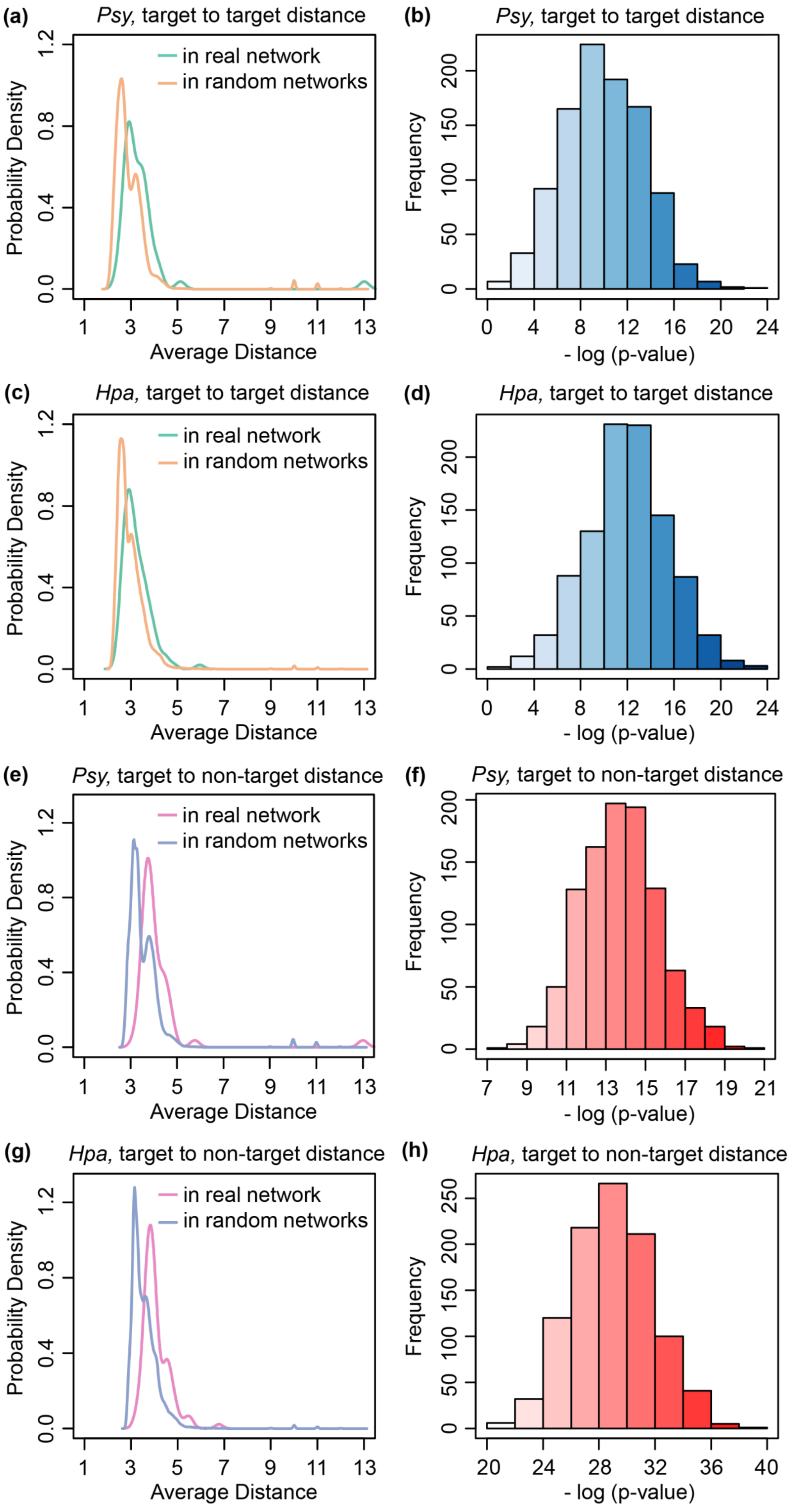
(a)
Psy, target to target distance
in real network
in random networks
Probability Density
Average Distance
(b)
Psy, target to target distance
Frequency
- log (p-value)
(c)
Hpa, target to target distance
in real network
in random networks
Probability Density
Average Distance
(d)
Hpa, target to target distance
Frequency
- log (p-value)
(e)
Psy, target to non-target distance
in real network
in random networks
Probability Density
Average Distance
(f)
Psy, target to non-target distance
Frequency
- log (p-value)
(g)
Hpa, target to non-target distance
in real network
in random networks
Probability Density
Average Distance
(h)
Hpa, target to non-target distance
Frequency
- log (p-value)

**Figure S2 The comparison of the distances between different types of proteins, between real and random networks.**

(a and c) The distributions of average distances from each of *Psy* (or *Hpa*) targets to the rest of *Psy* (or *Hpa*) targets, in the comprehensive *Arabidopsis* PPI network and 1,000 random *Arabidopsis* PPI networks. (b and d) Histograms showing the p-values resulted from the comparison of average distances from each of *Psy* (or *Hpa*) targets to the rest of *Psy* (or *Hpa*) targets, between the comprehensive *Arabidopsis* PPI network and each of 1,000 random *Arabidopsis* PPI networks. The p-values from one-tailed Wilcoxon's test are used. (e and g) The distributions of average distances from each of *Psy* (or *Hpa*) targets to non-*Psy* (or non-*Hpa*) targets, in the comprehensive *Arabidopsis* PPI network and 1,000 random *Arabidopsis* PPI networks. (f and h) Histograms showing the p-values resulted from the comparison of average distances from each of *Psy* (or *Hpa*) targets to non-*Psy* (or non-*Hpa*) targets, between the comprehensive *Arabidopsis* PPI network and each of 1,000 random *Arabidopsis* PPI networks. The p-values from one-tailed Wilcoxon's test are used.

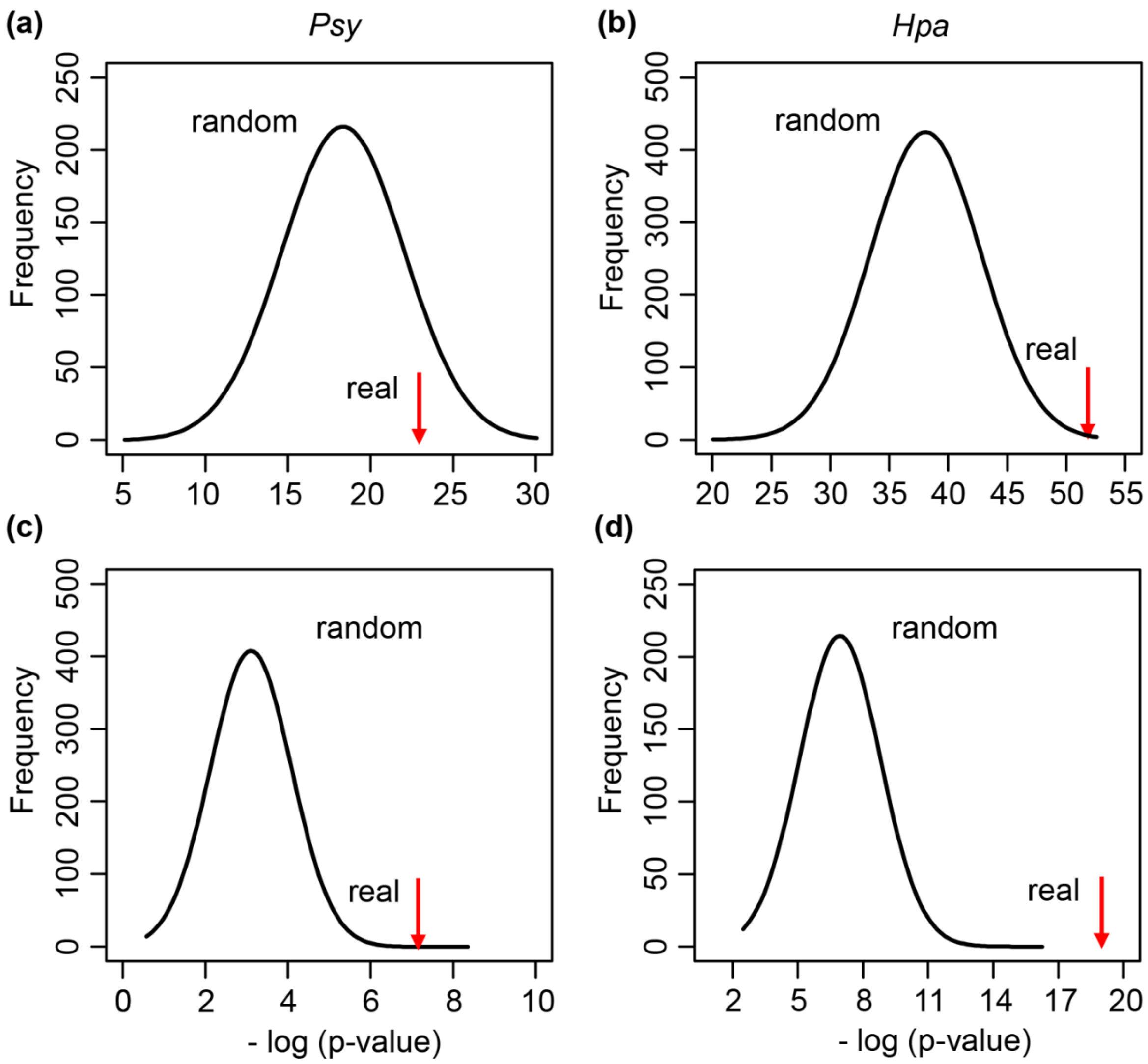


**Figure S3 The p-values for the comparison of the distances between different types of proteins, in the real PPI network and random networks, respectively.**

(a and b) For each network, the average distances from each of *Psy* (or *Hpa*) targets to the rest of *Psy* (or *Hpa*) targets and to non-*Psy* (or non-*Hpa*) targets are compared. The p-value from one-tailed Wilcoxon's test is used to estimate the significance of comparison. The distribution of p-values from 1,000 random networks (random) is plotted, while the p-value from the real PPI network (real) is indicated by the red arrow. (c and d) For each network, the average distances from each of *Psy* (or *Hpa*) targets to the effector targets interacting with the same *Psy* (or *Hpa*) effectors and to those interacting with different *Psy* (or *Hpa*) effectors are compared. The p-value from one-tailed Wilcoxon's test is used to estimate the significance of comparison. The distribution of p-values from 1,000 random networks (random) is plotted, while the p-value from the real PPI network (real) is indicated by the red arrow.

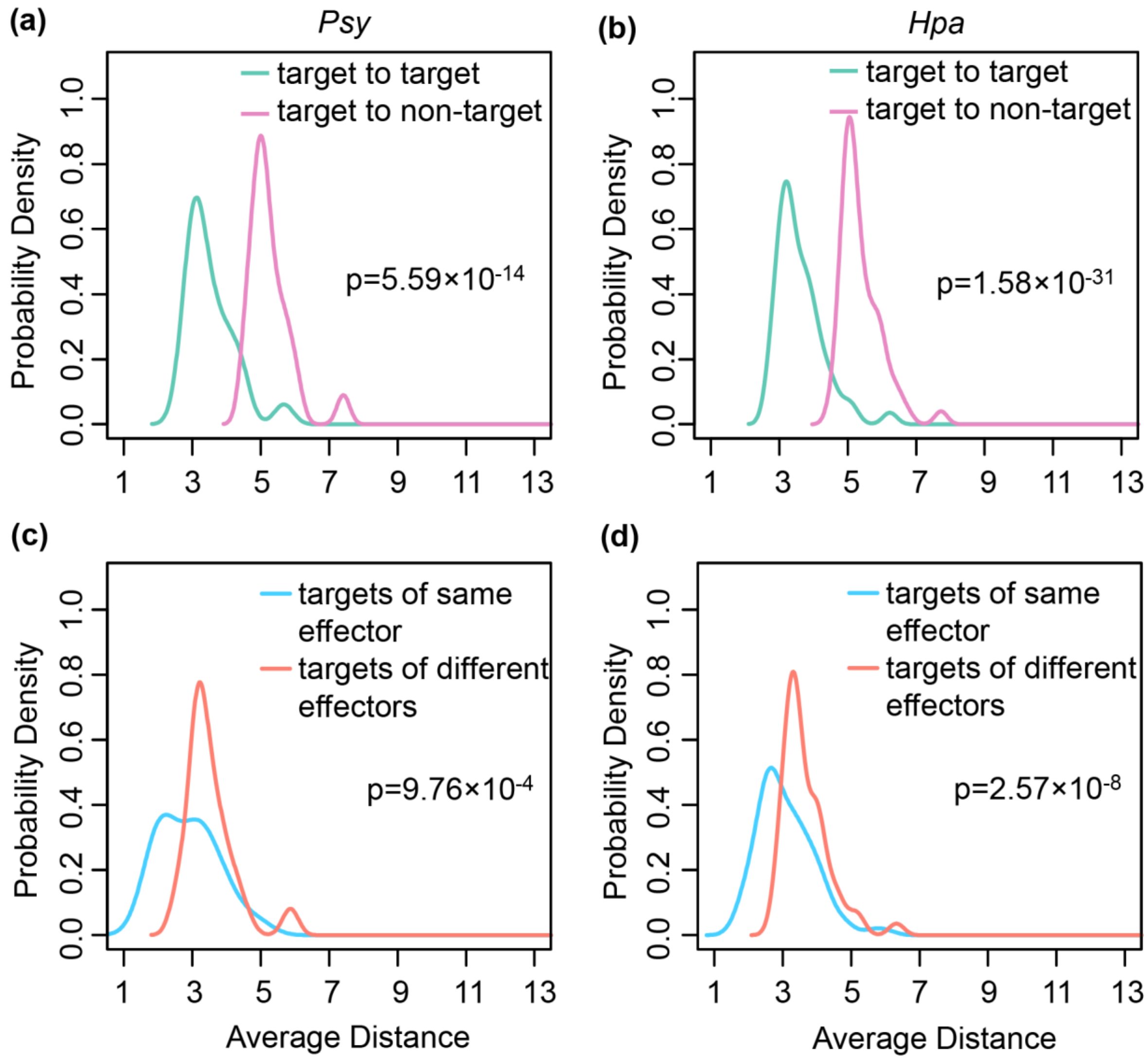


**Figure S4 The distributions of average distances between different types of proteins in AI-$1_{\mathrm{MAIN}}$.**

The distributions of average distances (a) from each of *Psy* targets to the rest of *Psy* targets and to the proteins not targeted by the *Psy* effectors; (b) from each of *Hpa* targets to the rest of *Hpa* targets and to the proteins not targeted by the *Hpa* effectors; (c) from each of *Psy* targets to the effector targets interacting with the same *Psy* effectors and to those interacting with different *Psy* effectors; (d) from each of *Hpa* targets to the effector targets interacting with the same *Hpa* effectors and to those interacting with different *Hpa* effectors in AI-$1_{\mathrm{MAIN}}$ are plotted. The significance of the difference in distance distributions is estimated using one-tailed Wilcoxon's test.

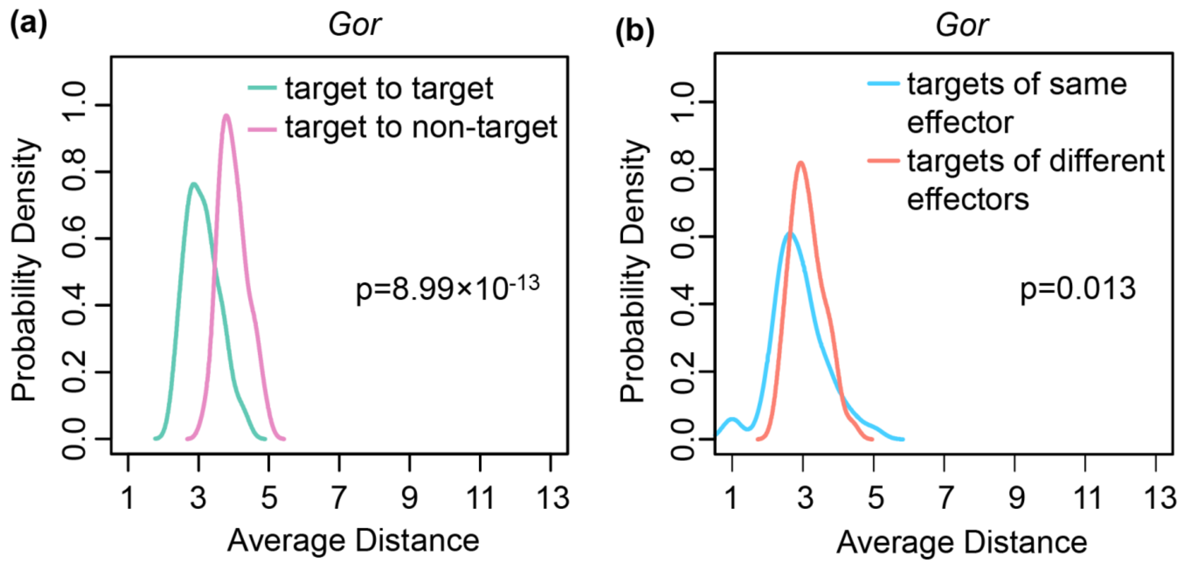


**Figure S5 The distributions of average distances between different types of proteins for *Gor* in the comprehensive *Arabidopsis* PPI network.**

The distributions of average distances (a) from each of *Gor* targets to the rest of *Gor* targets and to non-*Gor* targets; (b) from each of *Gor* targets to the effector targets interacting with the same *Gor* effectors and to those interacting with different *Gor* effectors in the comprehensive *Arabidopsis* PPI network are plotted. The significance of the difference in distance distributions is estimated using one-tailed Wilcoxon's test.

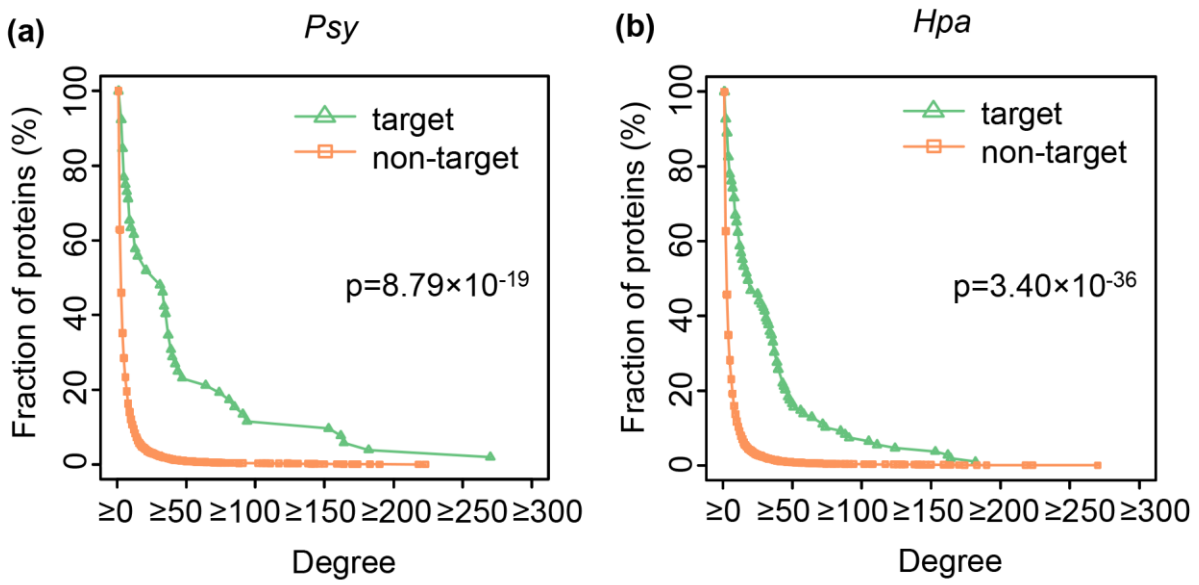


**Figure S6 The cumulative distributions of the degree of the effector targets and non-targets in the comprehensive *Arabidopsis* PPI network.**

The cumulative distributions of the degree of *Psy* targets, non-*Psy* targets, *Hpa* targets and non-*Hpa* targets in the comprehensive *Arabidopsis* PPI network are plotted after removing an outlier. The significance of the difference in degree distributions is estimated using one-tailed Wilcoxon's test.

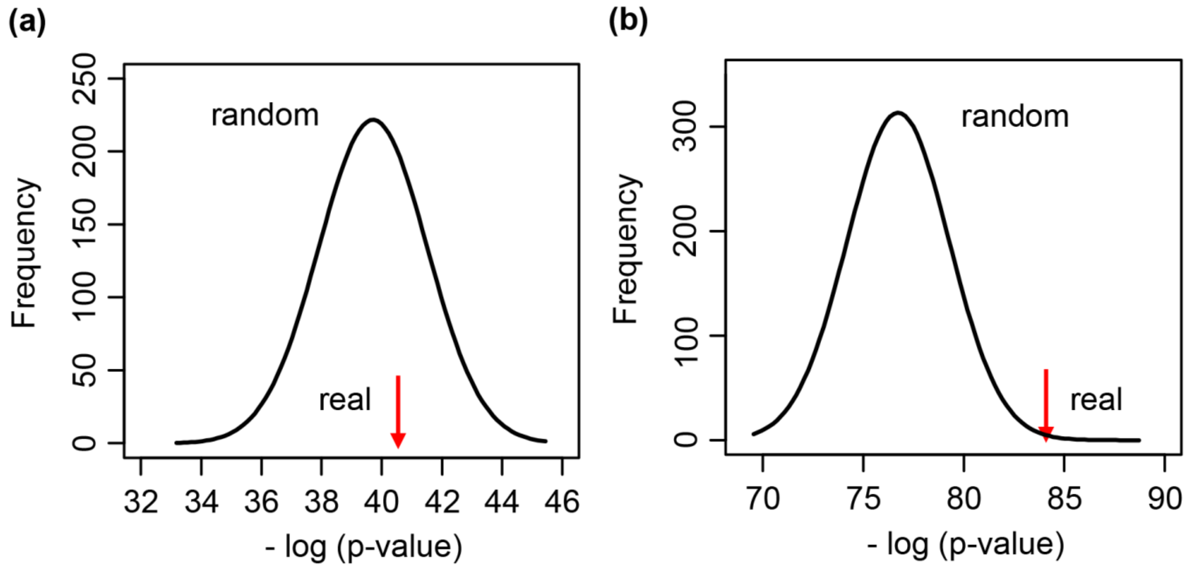


**Figure S7 The p-values for the comparison of betweenness centrality, in the real PPI network and random networks, respectively.**

For each network, the betweenness centrality of *Psy* (or *Hpa*) targets and non-*Psy* (or non-*Hpa*) targets are compared. The p-value from one-tailed Wilcoxon's test is used to estimate the significance of comparison. The distribution of p-values from 1,000 random networks (random) are plotted, while the p-value from the real PPI network (real) is indicated by the red arrow.

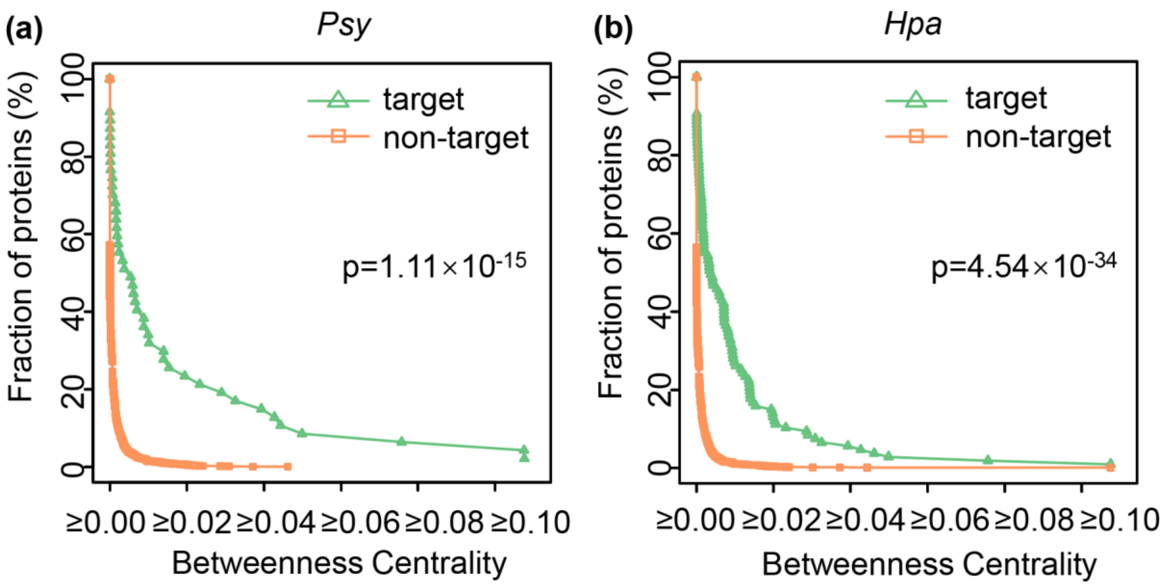


**Figure S8 The cumulative distributions of the betweenness centrality of the effector targets and non-targets in AI-1$_{\text{MAIN}}$.**

The cumulative distributions of the betweenness centrality of *Psy* targets, the proteins not targeted by the *Psy* effectors, *Hpa* targets and the proteins not targeted by the *Hpa* effectors AI-1$_{\text{MAIN}}$ are plotted. The significance of the difference in betweenness centrality distributions is estimated using one-tailed Wilcoxon's test.

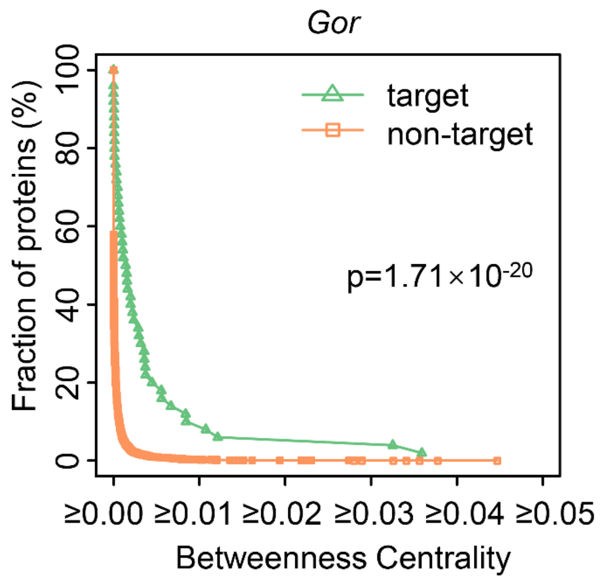


**Figure S9 The cumulative distributions of the betweenness centrality of the *Gor* targets and non-*Gor* targets in the comprehensive *Arabidopsis* PPI network.**

The significance of the difference in betweenness centrality distributions is estimated using one-tailed Wilcoxon's test. An outlier is removed from this figure.

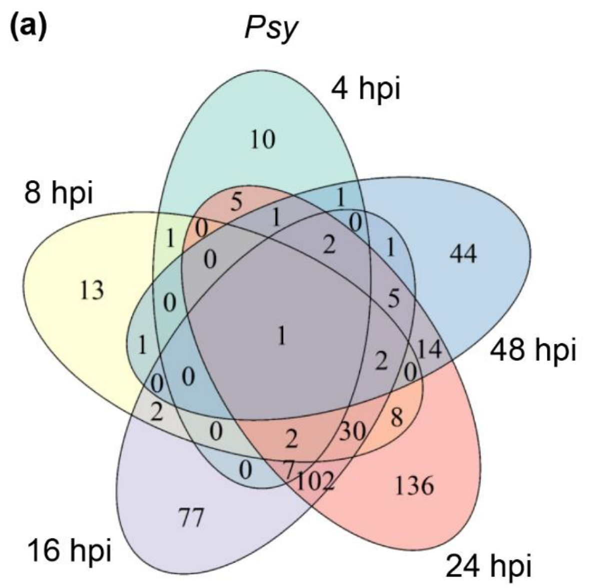


**Figure S10 Venn-diagrams depicting the number of overlapped DEGs between different time points.**

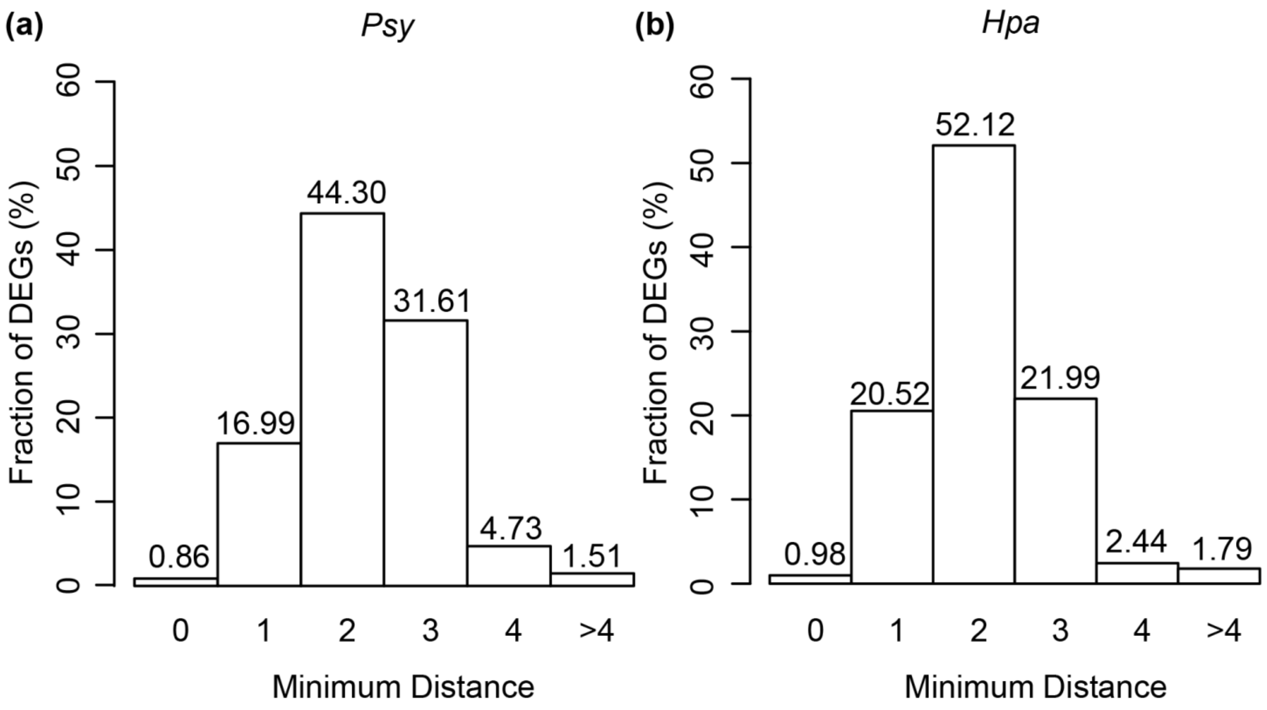


**Figure S11 The minimum distances from each of *Psy* (or *Hpa*) DEGs to *Psy* (or *Hpa*) targets in the comprehensive *Arabidopsis* PPI network.**

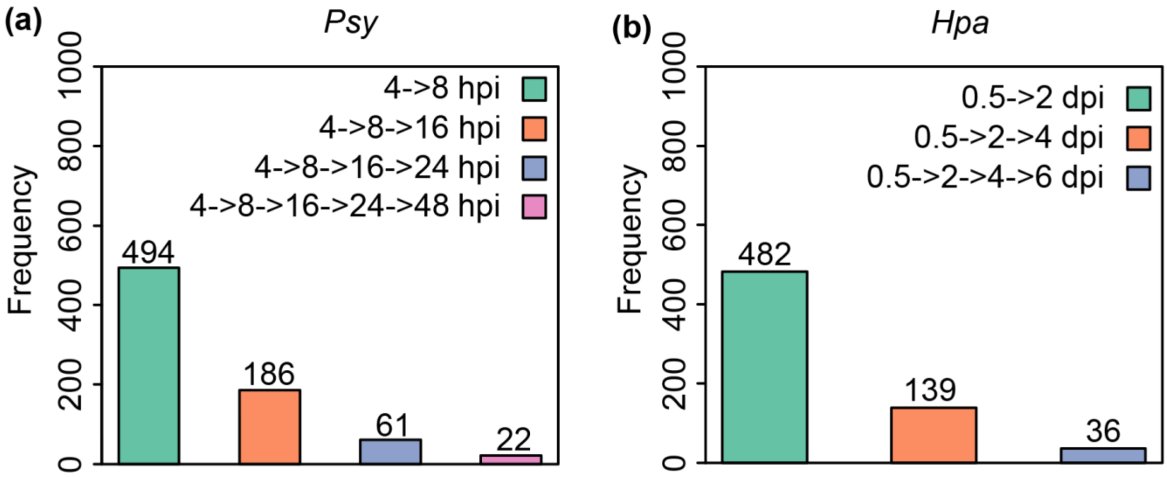


**Figure S12 The frequency for observing consistently increasing distances between random proteins and the effector targets in 1,000 random trials.**

For DEGs at each time-point, an equal number of random proteins are picked and their distances to the effector targets are calculated. Such random trial is repeated 1,000 times. The frequency of the random trials showing the gradually increasing distances from DEGs to the effector targets over the first two, three, four (and five) time points are plotted here.

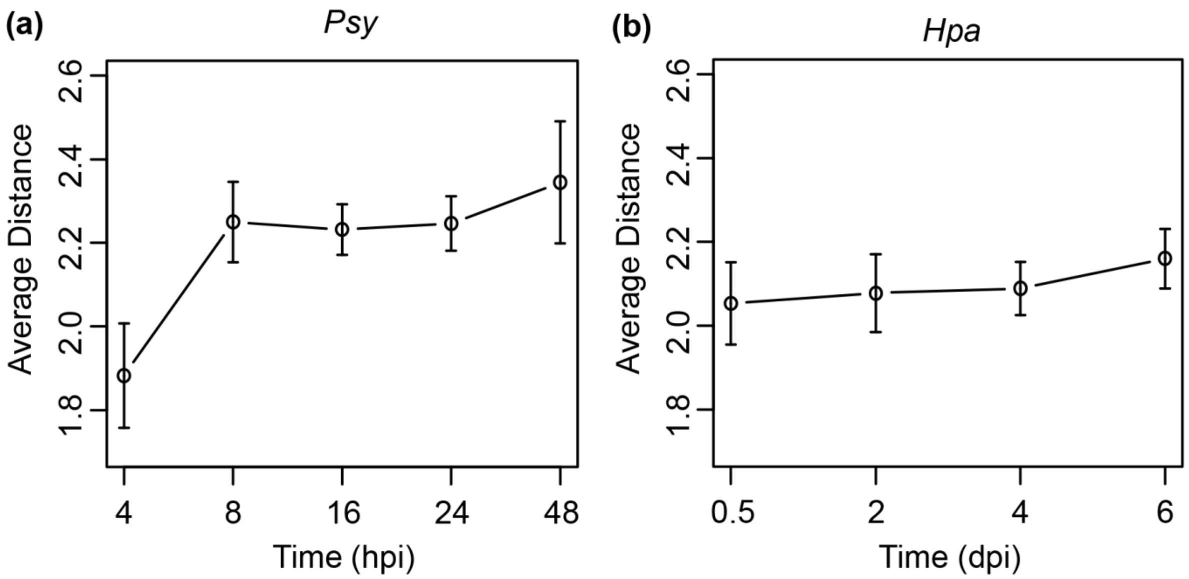


**Figure S13 The changes of the average distances from DEGs at different time points to the effector targets in a larger *Arabidopsis* network.**

The average distances from DEGs at each time points during *Psy* or *Hpa* infection to *Psy* or *Hpa* targets are plotted in a larger *Arabidopsis* network, which consists of the comprehensive *Arabidopsis* PPI network and *Arabidopsis* protein-DNA interactions. Error bars represent the standard errors of the average distances.

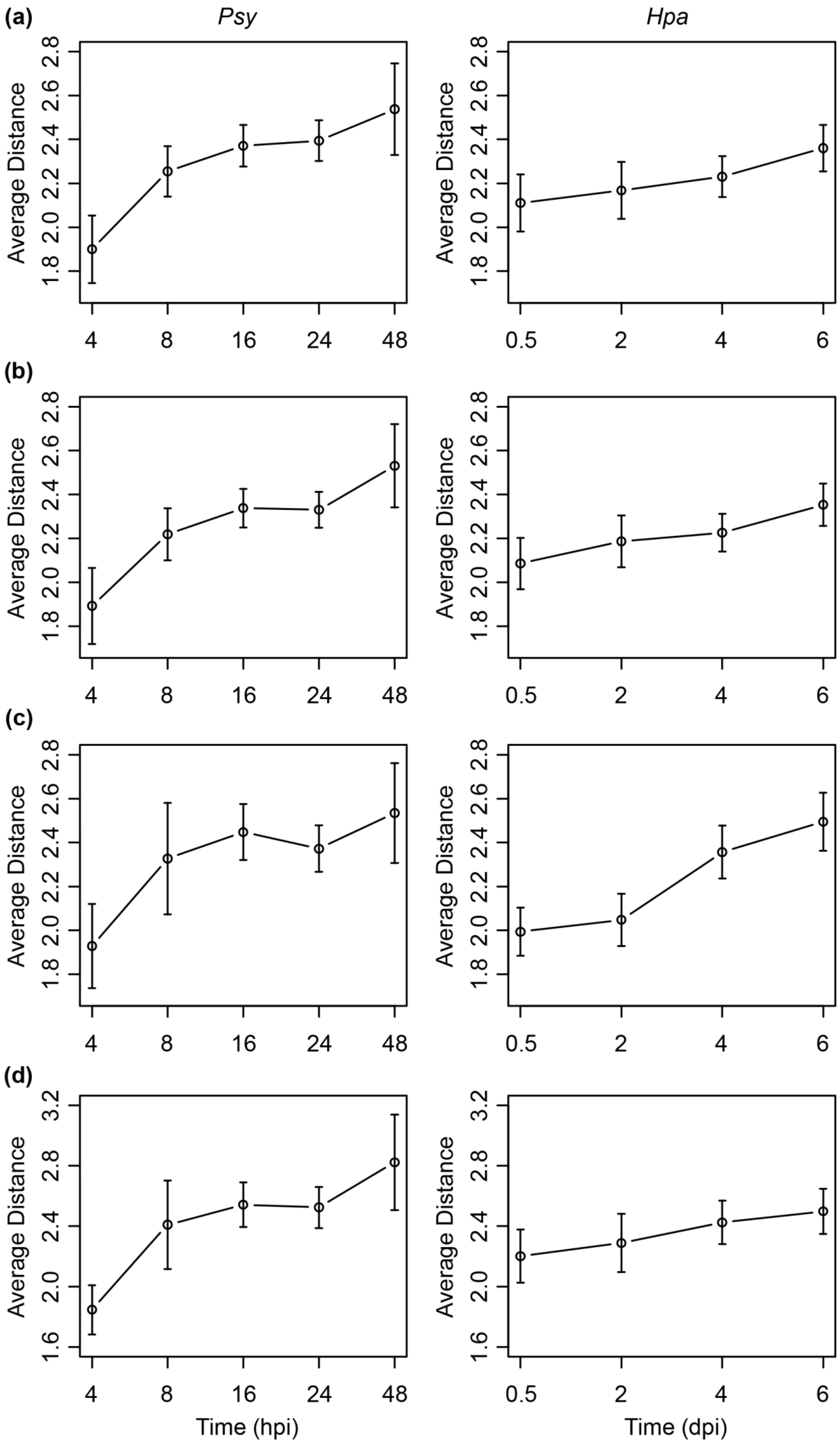
(a)
Psy
Hpa
(b)
(c)
(d)
Average Distance
1.8
2.0
2.2
2.4
2.6
2.8
1.6
3.2
4
8
16
24
48
0.5
2
6
Time (hpi)
Time (dpi)

**Figure S14 The changes of the average distances from DEGs at different time points to the effector targets after removing different proportions of PPIs.**

The average distances from DEGs at each time points during *Psy* or *Hpa* infection to *Psy* or *Hpa* targets are plotted when (a) 10%, (b) 20%, (c) 30% and (d) 40% PPIs are randomly removed from the comprehensive *Arabidopsis* PPI network. Error bars represent the standard errors of the average distances.

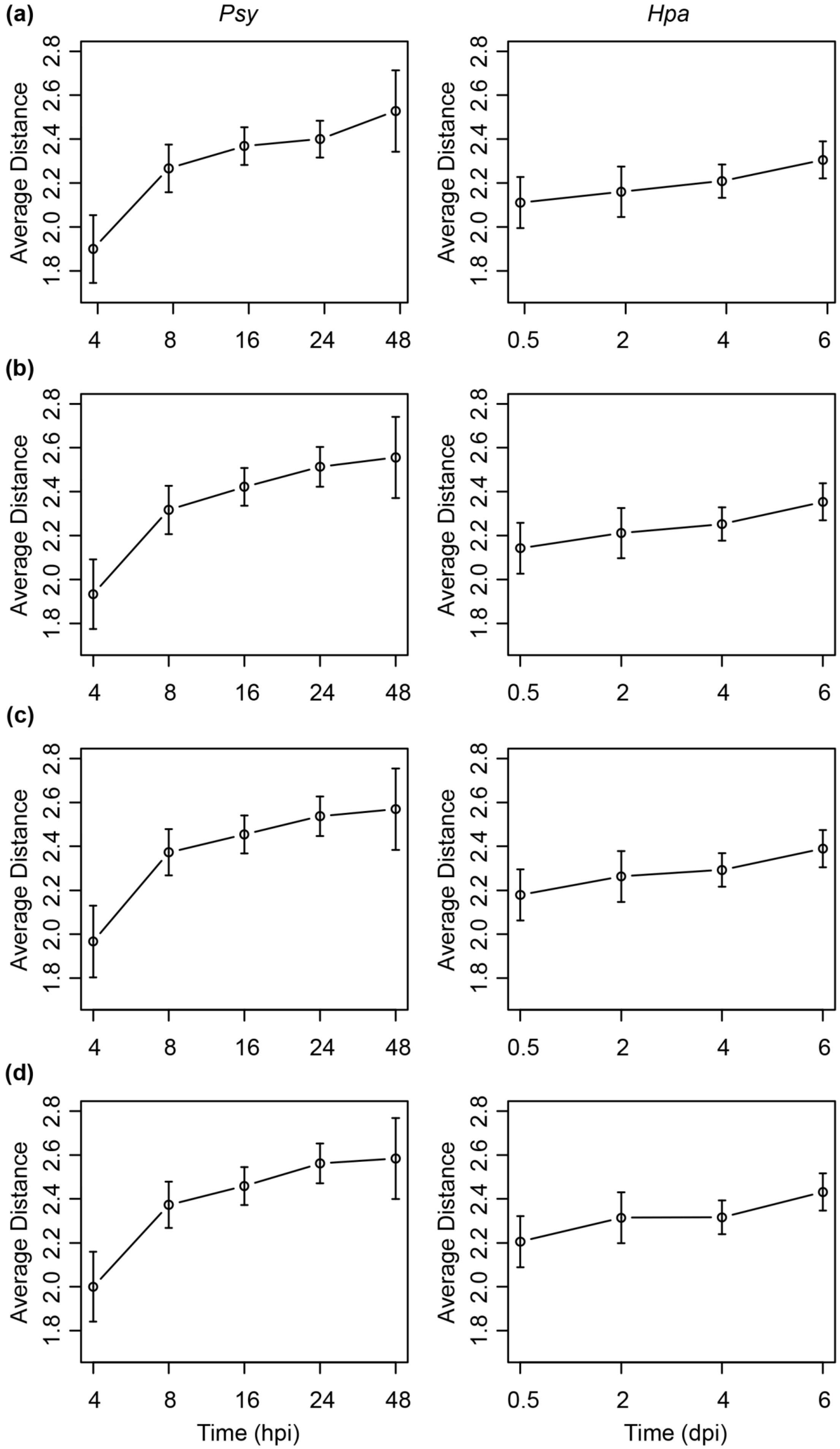

(a)
Psy
Hpa
Average Distance
1.8 2.0 2.2 2.4 2.6 2.8
4 8 16 24 48
0.5 2 4 6
(b)
(c)
(d)
Time (hpi)
Time (dpi)

**Figure S15 The changes of the average distances from DEGs at different time points to the rest of effector targets after removing different proportions of effector targets.**

When (a) 10%, (b) 20%, (c) 30% and (d) 40% *Psy* or *Hpa* targets are randomly removed from the comprehensive *Arabidopsis* PPI network, the average distances from DEGs at each time points during *Psy* or *Hpa* infection to the rest of *Psy* or *Hpa* targets are plotted. Error bars represent the standard errors of the average distances.

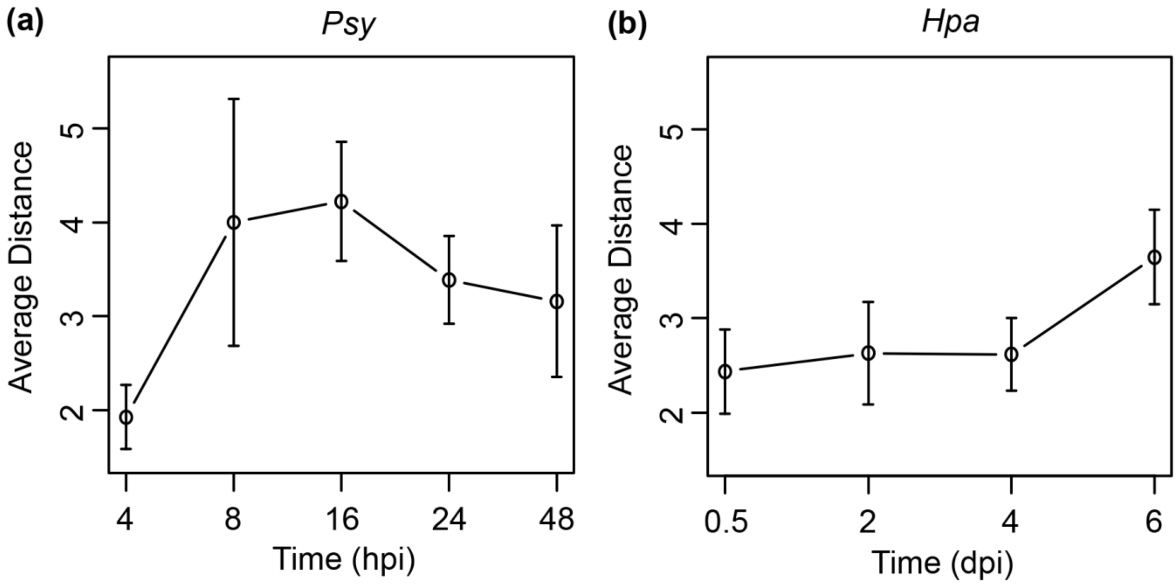


**Figure S16 The changes of the average distances from DEGs at different time points to the effector targets in AI-1$_{\mathrm{MAIN}}$.**

The average distances from DEGs at each time points during *Psy* or *Hpa* infection to *Psy* or *Hpa* targets in AI-1$_{\mathrm{MAIN}}$ are plotted. Error bars represent the standard errors of the average distances.

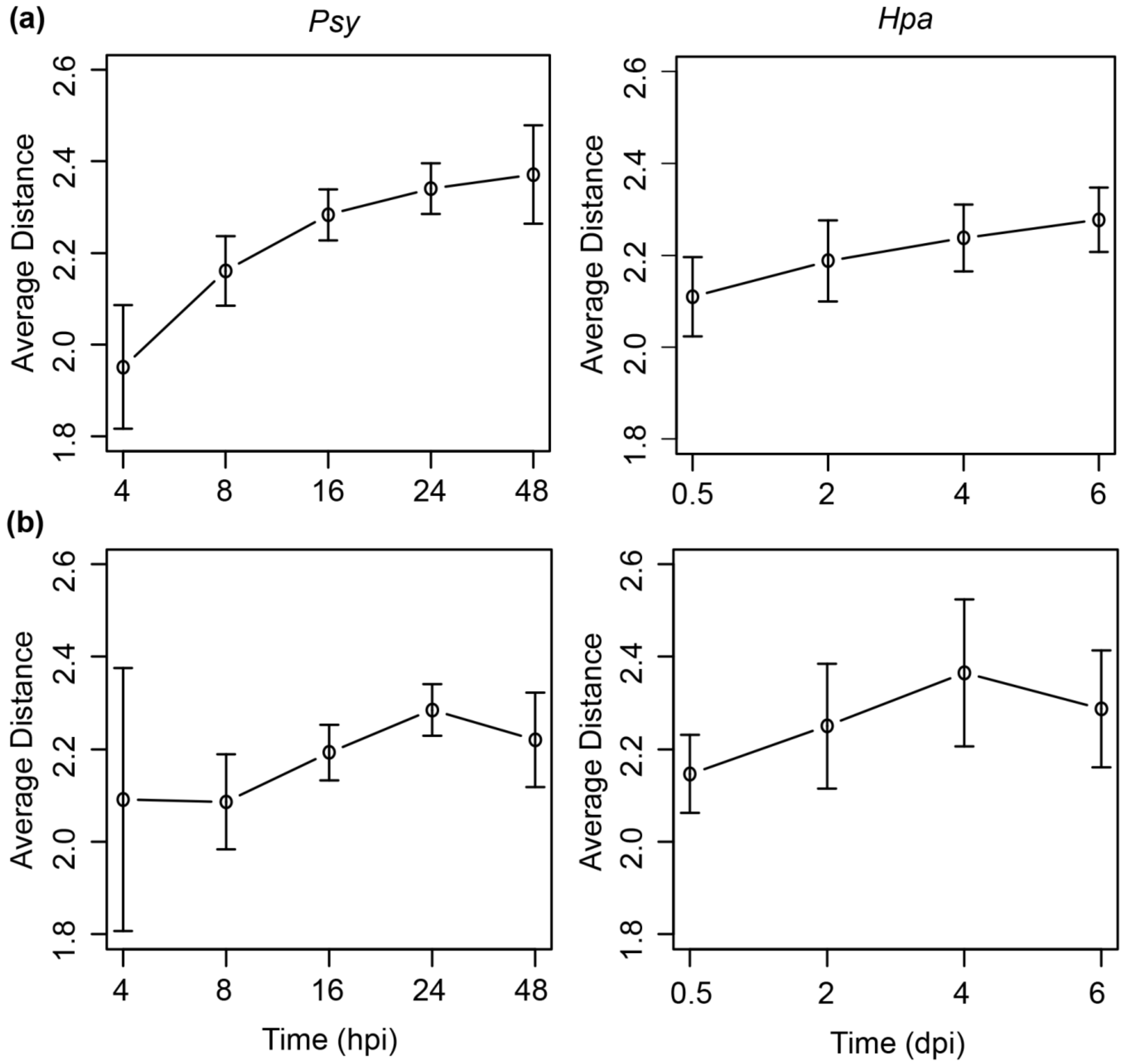


**Figure S17 The changes of the average distances for different types of DEGs at different time points to the effector targets.**

The average distances (a) from all DEGs (including up-regulated and down-regulated DEGs) at each time points during *Psy* or *Hpa* infection to *Psy* or *Hpa* targets; (b) from down-regulated DEGs at each time points during *Psy* or *Hpa* infection to *Psy* or *Hpa* targets in the comprehensive *Arabidopsis* PPI network are plotted. Error bars represent the standard errors of the average distances.

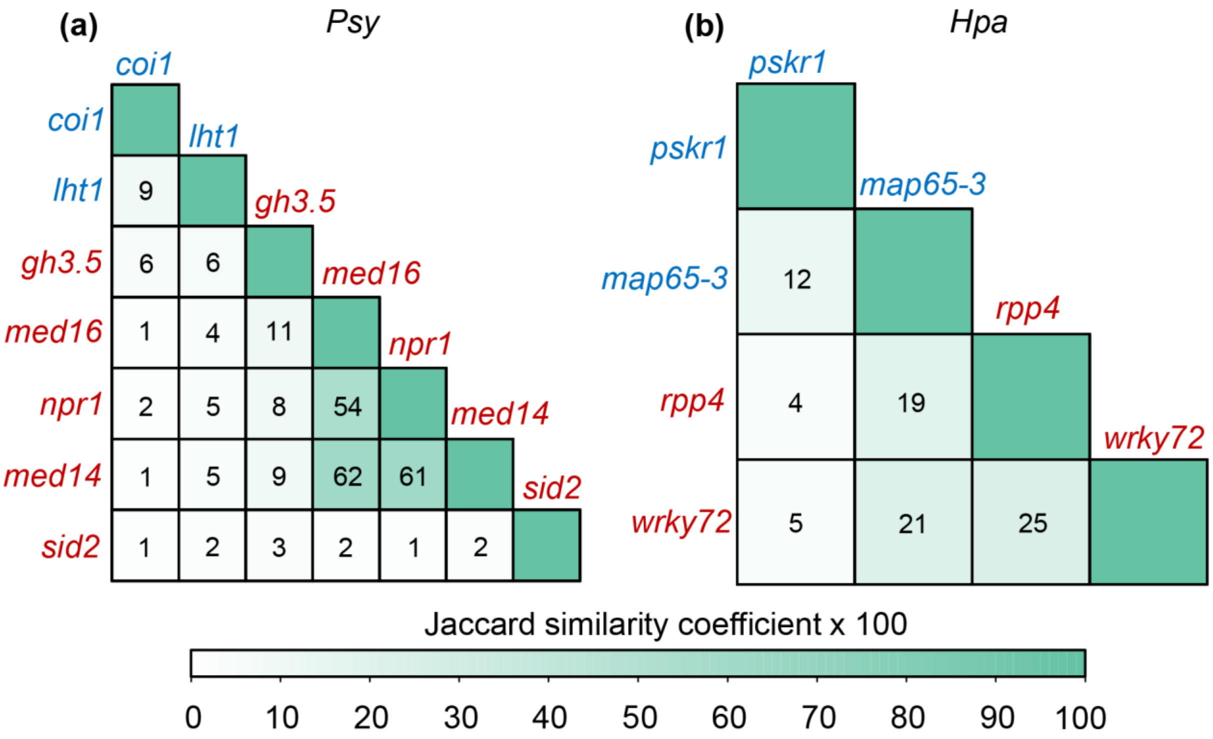


**Figure S18 The similarities between DEGs in different mutants using different definitions of DEGs.** The DEGs are obtained by comparing mutants inoculated with *Psy* or *Hpa* versus mutants treated with mock control. The similarity between two DEG sets in different mutants in the context of (a) *Psy* or (b) *Hpa* infection is estimated by Jaccard similarity coefficient. The Jaccard similarity coefficient is calculated by taking the number of DEGs involved in both of the two sets divided by the number of DEGs involved in either of the two sets. The value marked in the cells is the product of the Jaccard similarity coefficient and 100.

**Table S1 The comparison of the betweenness centrality distributions between the effector targets and non-targets among hubs.**

| Hub[a] | 5 | 10 | 15 | 20 |
|---|---|---|---|---|
| *Psy*[b] | $5.2\times10^{-12}$ | $1.2\times10^{-8}$ | $1.1\times10^{-6}$ | $2.3\times10^{-4}$ |
| *Hpa*[b] | $8.1\times10^{-20}$ | $2.1\times10^{-13}$ | $8.5\times10^{-9}$ | $1.4\times10^{-5}$ |

[a]The proteins, whose degrees are more than 5, 10, 15 and 20, in the comprehensive *Arabidopsis* PPI network are defined as hubs.

[b]The significance of the difference in betweenness centrality distributions between hubs belonging to *Psy* targets and non-*Psy* targets (or belonging to *Hpa* targets and non-*Hpa* targets) is estimated using one-tailed Wilcoxon's test. The p-value is shown in the table.

**Table S2 The number of DEGs at different time points during infection in the comprehensive *Arabidopsis* PPI network.**

| | *Psy* infection | | | | | *Hpa* infection | | | |
|---|---|---|---|---|---|---|---|---|---|
| Time (hpi/dpi) | 4 | 8 | 16 | 24 | 48 | 0.5 | 2 | 4 | 6 |
| # DEG | 30 | 60 | 231 | 315 | 72 | 190 | 156 | 303 | 335 |

Only the up-regulated genes included in the networks are considered.

**Table S3 The number of DEGs at different time points during infection in AI-1$_{MAIN}$.**

| | *Psy* infection | | | | | *Hpa* infection | | | |
|---|---|---|---|---|---|---|---|---|---|
| Time (hpi/dpi) | 4 | 8 | 16 | 24 | 48 | 0.5 | 2 | 4 | 6 |
| # DEG | 14 | 18 | 72 | 98 | 25 | 73 | 46 | 91 | 102 |

Only the up-regulated genes included in the networks are considered.

**Table S4 The comparison of the average distances between DEGs of two adjacent time points before and after removing the effector targets from AI-1$_{\text{MAIN}}$.**

| | *Psy* infection | | | | *Hpa* infection | | |
|---|---|---|---|---|---|---|---|
| Time[a] | 4hpi->8hpi | 8hpi->16hpi | 26hpi->24hpi | 24hpi->48hpi | 0.5dpi->2dpi | 2dpi->4dpi | 4dpi->6dpi |
| + targets[b] | 4.278 | 4.569 | 1.663 | 2.120 | 2.565 | 2.308 | 2.441 |
| - targets[c] | 5.278 | 5.958 | 3.367 | 2.720 | 3.913 | 4.978 | 3.725 |

[a]Two adjacent time points during *Psy* or *Hpa* infection.

[b]The average distances between DEGs of two adjacent time points when effector targets are included AI-1$_{\text{MAIN}}$.

[c]The average distances between DEGs of two adjacent time points after removing *Psy* or *Hpa* targets from AI-1$_{\text{MAIN}}$.

**Table S5 The average distances from the DEGs in susceptible or resistant mutants to the effector targets after removing different proportions of PPIs.**

| Mutated gene[a] | Phenotype | Raw Network[b] | | Remove 10% PPIs[c] | | Remove 20% PPIs[c] | | Remove 30% PPIs[c] | | Remove 40% PPIs[c] | |
|---|---|---|---|---|---|---|---|---|---|---|---|
| | | Distance | Rank | Distance | Rank | Distance | Rank | Distance | Rank | Distance | Rank |
| *Psy* infection | | | | | | | | | | | |
| *wrky 18/40* | resistant | 2.292 | 1 | 2.422 (0.058) | 3 | 2.430 (0.051) | 3 | 2.457 (0.076) | 3 | 2.497 (0.106) | 3 |
| *ein2* | resistant | 2.249 | 2 | 2.473 (0.017) | 1 | 2.474 (0.039) | 1 | 2.486 (0.078) | 1 | 2.515 (0.097) | 1 |
| *coi1* | resistant | 2.214 | 3 | 0.342 (0.034) | 5 | 2.361 (0.037) | 5 | 2.397 (0.076) | 5 | 2.442 (0.106) | 5 |
| *lht1* | resistant | 2.206 | 4 | 2.381 (0.048) | 4 | 2.400 (0.053) | 4 | 2.444 (0.089) | 4 | 2.496 (0.127) | 4 |
| *pad4* | susceptible | 2.205 | 5 | 2.340 (0.014) | 6 | 2.351 (0.022) | 7 | 2.381 (0.053) | 7 | 2.416 (0.084) | 7 |
| *npr1* | susceptible | 2.198 | 6 | 2.339 (0.034) | 7 | 2.351 (0.034) | 6 | 2.384 (0.061) | 6 | 2.421 (0.089) | 6 |
| *pad2* | susceptible | 2.176 | 7 | 2.253 (0.030) | 8 | 2.268 (0.038) | 8 | 2.295 (0.066) | 8 | 2.321 (0.096) | 8 |
| *sid2* | susceptible | 2.175 | 8 | 2.446 (0.040) | 2 | 2.448 (0.043) | 2 | 2.480 (0.075) | 2 | 2.501 (0.084) | 2 |

| Mutated gene[a] | Phenotype | Raw Network[b] | | Remove 10% PPIs[c] | | Remove 20% PPIs[c] | | Remove 30% PPIs[c] | | Remove 40% PPIs[c] | |
|---|---|---|---|---|---|---|---|---|---|---|---|
| | | Distance | Rank | Distance | Rank | Distance | Rank | Distance | Rank | Distance | Rank |
| *Hpa* infection | | | | | | | | | | | |
| *map65-3* | resistant | 2.122 | 1 | 2.389 (0.076) | 2 | 2.355 (0.081) | 2 | 2.373 (0.140) | 2 | 2.358 (0.166) | 2 |
| *pskr1* | resistant | 2.036 | 2 | 2.457 (0.146) | 1 | 2.444 (0.110) | 1 | 2.461 (0.219) | 1 | 2.509 (0.342) | 1 |
| *wrky72* | susceptible | 2.000 | 3 | 2.030 (0.041) | 4 | 2.031 (0.058) | 4 | 2.070 (0.084) | 4 | 2.133 (0.171) | 4 |
| *rpp4* | susceptible | 2.000 | 4 | 2.070 (0.127) | 3 | 2.108 (0.158) | 3 | 2.128 (0.184) | 3 | 2.170 (0.226) | 3 |

[a]Mutants are more resistant or susceptible (column 2) to *Psy* or *Hpa* compared with wild-types.

[b]The average distances between the DEGs and *Psy* or *Hpa* targets in the comprehensive Arabidopsis PPI network. The corresponding distances are ranked in descending order.

[c]The average distances between the DEGs and *Psy* or *Hpa* targets after different proportions of PPIs are randomly removed from the comprehensive Arabidopsis PPI network. The value marked in the parenthesis is the standard deviation of the average distances between the DEGs and *Psy* or *Hpa* targets from 10 random trials.

**Table S6 The average distances from the DEGs in susceptible or resistant mutants to the effector targets in AI-1$_{\mathrm{MAIN}}$.**

| Series | Platform | Mutated gene[a] | Phenotype | # DEG[b] | Distance[c] | Rank[d] |
|---|---|---|---|---|---|---|
| *Psy* infection | | | | | | |
| GSE6829 | GPL198 | *wrky18/40* | resistant | 57 | 4.105 | 1 |
| GSE19109 | GPL198 | *lht1* | resistant | 231 | 3.853 | 2 |
| GSE18978 | GPL198 | *ein2* | resistant | 63 | 3.571 | 3 |
| GSE18978 | GPL198 | *npr1* | susceptible | 200 | 3.545 | 4 |
| GSE18978 | GPL198 | *pad2* | susceptible | 76 | 3.526 | 5 |
| GSE18978 | GPL198 | *pad4* | susceptible | 349 | 3.450 | 6 |
| GSE18978 | GPL198 | *sid2* | susceptible | 139 | 3.374 | 7 |
| GSE18978 | GPL198 | *coi1* | resistant | 223 | 3.215 | 8 |
| *Hpa* infection | | | | | | |
| GSE22274 | GPL198 | *rpp4* | susceptible | 20 | 6.350 | 1 |
| GSE73351 | GPL198 | *map65-3* | resistant | 22 | 3.136 | 2 |
| GSE37255 | GPL198 | *pskr1* | resistant | 9 | 2.667 | 3 |
| GSE18329 | GPL198 | *wrky72* | susceptible | 24 | 2.208 | 4 |

[a]Mutants are more resistant or susceptible (column 4) to *Psy* or *Hpa* compared with wild-types.

[b]The number of genes differentially expressed in mutants (column 3) versus the corresponding wild-types, after inoculation with *Psy* or *Hpa*.

[c]The average distances between the DEGs and *Psy* or *Hpa* targets AI-1$_{\mathrm{MAIN}}$.

[d]The distances (column 6) are ranked in descending order.

**Table S7 The average distances from the DEGs in susceptible or resistant mutants to the effector targets using different definitions of DEGs in the comprehensive *Arabidopsis* PPI network.**

| Series | Platform | Mutated gene[a] | Phenotype | # DEG[b] | Distance[c] | Rank[d] |
|---|---|---|---|---|---|---|
| *Psy* infection | | | | | | |
| GSE11009 | GPL3638 | *coi1* | resistant | 80 | 2.325 | 1 |
| GSE19109 | GPL198 | *lht1* | resistant | 80 | 2.316 | 2 |
| GSE6556 | GPL198 | *gh3.5* | susceptible | 982 | 2.224 | 3 |
| GSE45214 | GPL12621 | *med16* | susceptible | 397 | 2.218 | 4 |
| GSE45214 | GPL12621 | *npr1* | susceptible | 248 | 2.208 | 5 |
| GSE45214 | GPL12621 | *med14* | susceptible | 276 | 2.180 | 6 |
| GSE40544 | GPL13294 | *sid2* | susceptible | 67 | 2.164 | 7 |
| *Hpa* infection | | | | | | |
| GSE37255 | GPL198 | *pskr1* | resistant | 62 | 2.200 | 1 |
| GSE73351 | GPL198 | *map65-3* | resistant | 319 | 2.143 | 2 |
| GSE22274 | GPL198 | *rpp4* | susceptible | 369 | 2.120 | 4 |
| GSE18329 | GPL198 | *wrky72* | susceptible | 910 | 2.102 | 3 |

[a]Mutants are more resistant or susceptible (column 4) to *Psy* or *Hpa* compared with wild-types.

[b]The number of genes differentially expressed in mutants (column 3) with *Psy* or *Hpa* inoculation versus mock control.

[c]The average distances between the DEGs and *Psy* or *Hpa* targets in the comprehensive *Arabidopsis* PPI network.

[d]The distances (column 6) are ranked in descending order.
